# Kinetics Control of Mithrene Formation in a High-Pressure Inert Environment: A Robust Solvent-Free Route to Superior-Quality Films

**DOI:** 10.1021/acsami.5c15192

**Published:** 2025-11-03

**Authors:** Seunghwan Kim, Kitae Kim, Aelim Ha, Eunki Yoon, Sooyeon Pak, Eunjong Yoo, Ki Hoon Nam, Seung Min Kwak, Won Kook Choi, Young Yong Kim, Kyu Hyoung Lee, Yeonjin Yi, Soohyung Park

**Affiliations:** † Advanced Analysis and Data Center, 58975 Korea Institute of Science and Technology (KIST), Seoul 02792, Republic of Korea; ‡ Department of Physics, 26721 Yonsei University, 50 Yonsei-Ro, Seodaemun-Gu, Seoul 03722, Republic of Korea; § Department of Materials Science and Engineering, 26721 Yonsei University, Seoul 03722, Republic of Korea; ∥ Micro/Nano Fabrication Center, KIST, Seoul 02792, Republic of Korea; ⊥ Center for Opto-Electronic Materials and Devices, KIST, Seoul 02792, Republic of Korea; # Beamline Division, Pohang Accelerator Laboratory, 34995 POSTECH, Pohang 37673, Republic of Korea; ∇ Division of Nano & Information Technology, KIST School, University of Science and Technology (UST), Seoul 02792, Republic of Korea

**Keywords:** metal organic chalcogenolates (MOCs), mithrene, solvent-free synthesis, quasi-two-dimensional materials, reaction kinetics control

## Abstract

Metal organic chalcogenolates (MOCs) constitute a promising class of materials for optoelectronic applications owing to their unique 2D layered hybrid structure and inherent environmental stability. Among these materials, mithrene (silver phenylselenolate, AgSePh) is particularly compelling because of its sharp blue emission and notable anisotropic excitonic properties. However, conventional solvent-assisted mithrene synthesis methods are often associated with the introduction of chemical complexities as well as compromised film quality. Addressing these limitations, this study provides a robust solvent-free strategy for synthesizing high-quality mithrene thin films through precise pressure and temperature control in an inert gas environment, leading to optimized reaction kinetics. Comprehensive characterization through grazing incidence wide-angle X-ray scattering (GIWAXS), scanning electron microscopy (SEM), X-ray photoelectron spectroscopy (XPS), UV–vis absorption, and photoluminescence (PL) spectroscopy revealed that the resulting films possess greatly improved crystallinity, enhanced excitonic absorption, and significantly greater PL emission than their solvent-processed counterparts. Notably, a previously unreported excitonic feature (X_α_) was identified, possibly originating from the high structural coherence along the out-of-plane direction achieved through our method. This study not only provides an advanced solvent-free route for high-quality MOC thin film fabrication but also unlocks avenues for their broader integration into next-generation optoelectronic devices, semiconductors, and catalysts.

## Graphical abstract

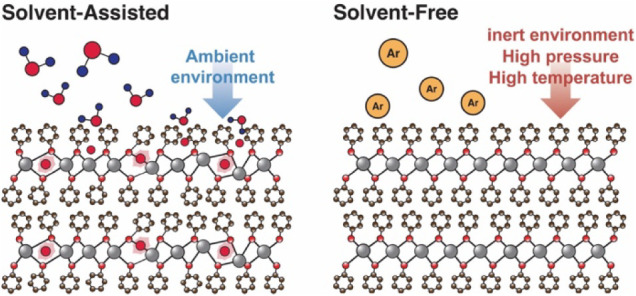

## 1 Introduction

Quasi-two-dimensional (quasi-2D) semiconductor materials are rapidly emerging as a compelling class of systems that retain the superior electrical and optical properties of low-dimensional 2D materials while overcoming the limitations imposed by the ultrathin nature of conventional 2D counterparts owing to their larger thicknesses.^1–5^ Among these materials, 2D metal organic chalcogenolates (MOCs) are particularly characterized by their outstanding optoelectronic properties. Consequently, 2D MOCs are being actively explored as highly promising candidates for next-generation optoelectronic devices, including photodetectors and light-emitting diodes.^6–8^

Within the 2D MOC family, silver phenylselenolate (AgSePh), also referred to as mithrene, serves as a prototypical example that clearly demonstrates the key low-dimensional characteristics of quasi-2D semiconductors. Mithrene exhibits unique optical properties, including anisotropic multiple exciton channels at room temperature, high exciton binding energy, and intense sharp blue emission.^9–11^ Further, it is extensively being studied as a representative excitonic platform for many-body physics research, and is showing significant promise in light harvesting and UV detection.^12–14^ These remarkable characteristics are a direct consequence of its distinct hybrid quantum well structure, in which inorganic and organic layers are alternately stacked, thus forming a highly ordered 2D framework.^15–19^ Therefore, the synthesis of high-quality, well-crystallized 2D MOC films, especially mithrene, is very important to fully exploit their exceptional optoelectronic performance and unlock their technological potential.

The pursuit of samples has led to investigations into various synthetic routes such as biphasic growth, sol–gel dip coating, and tarnishing method.^10,20–26^ Among these methods, the corrosion-like tarnishing method, which involves heating thermally evaporated silver (Ag) films in diphenyl diselenide (DPSe) vapor with assistant solvents, has been reported as a highly successful technique for producing large-area, high-quality crystalline mithrene thin films.^20,24^ Subsequent studies have highlighted that assistant solvents play an essential role in shaping the crystal conversion process during the implementation of this tarnishing method, with the choice of assistant solvent critically influencing the final sample quality. For example, Paritmongkol et al. systematically demonstrated this solvent dependency by fabricating crystals using solvents with varying polarities, boiling points, and functional groups.^8^ Despite such advancements in solvent-assisted approaches, significant limitations persist. These methods often require prolonged reaction times, potentially extending over several days (see the detail in Table S1).^8,20^ Furthermore, commonly employed solvents such as dimethyl sulfoxide (DMSO) and n-propylamine (PrNH_2_), while enhancing crystallinity, are associated with toxicity and volatility, which raise safety concerns during processing.^27–30^

In an attempt to overcome these solvent-related drawbacks, Maserati et al. proposed an alternative, faster solvent-free synthesis using oxidized silver films and phenylselenol (PhSeH) vapor, which reduced the reaction time to several minutes.^6,7,22^ However, this strategy mainly yields small nanocrystals (approximately 200 nm), thereby limiting film uniformity and adversely affecting electronic performance in device applications.^6^ Additionally, the inherent toxicity and air sensitivity of the PhSeH precursor in turn introduce significant challenges that require careful consideration.^20,31^

Building on these prior attempts, we here propose a novel solvent-free tarnishing methodology. The core of this approach is a custom-designed hermetically sealed (with an O-ring) stainless-steel chamber, which enables precise modulation of the internal vapor pressure via temperature control. This system affords superior control over the reaction kinetics and provides an inert argon (Ar) atmosphere, thereby minimizing contamination during synthesis. Consequently, this method effectively circumvents the solvent dependency inherent in previous processes, facilitating the direct synthesis of high quality thin films. Moreover, systematic tuning of reaction kinetics facilitates the identification of optimal pressure and temperature conditions, yielding 2D MOCs characterized by high crystallinity and excellent optical properties. This developed methodology could not only advance the fabrication of materials for mithrene-based optoelectronic devices but also provide a versatile synthetic platform for other 2D MOCs, including AuSePh and CuSePh, thus creating avenues toward an expanded family of hybrid quantum well materials.

## 2 Results and Discussion

### 2.1 Design and Validation of the High-Pressure Inert Environment Reaction System

The synthesis of high-quality 2D MOCs necessitates rigorous control over the reaction conditions, particularly minimizing contamination and enabling precise manipulation of the reaction kinetics. To meet these demands, we developed a custom-designed high-pressure inert environment reaction system, as shown in Figure 1. The core of the system is a stainless-steel chamber that is hermetically sealed with an O-ring to ensure complete isolation from the ambient atmosphere. Sample preparation, involving loading the Ag film and diphenyl-diselenide (DPSe) powder into this sealed chamber, is performed in a glovebox under an argon (Ar) atmosphere (Figure 1a). This approach contrasts sharply with preparations in air (Figure 1b), where materials are inevitably exposed to detrimental moisture, oxygen, and particulate contaminants. More detailed information on the sample preparation is provided in Figure S1 and Section 4.1 of the Experimental Details.

**Figure 1:**
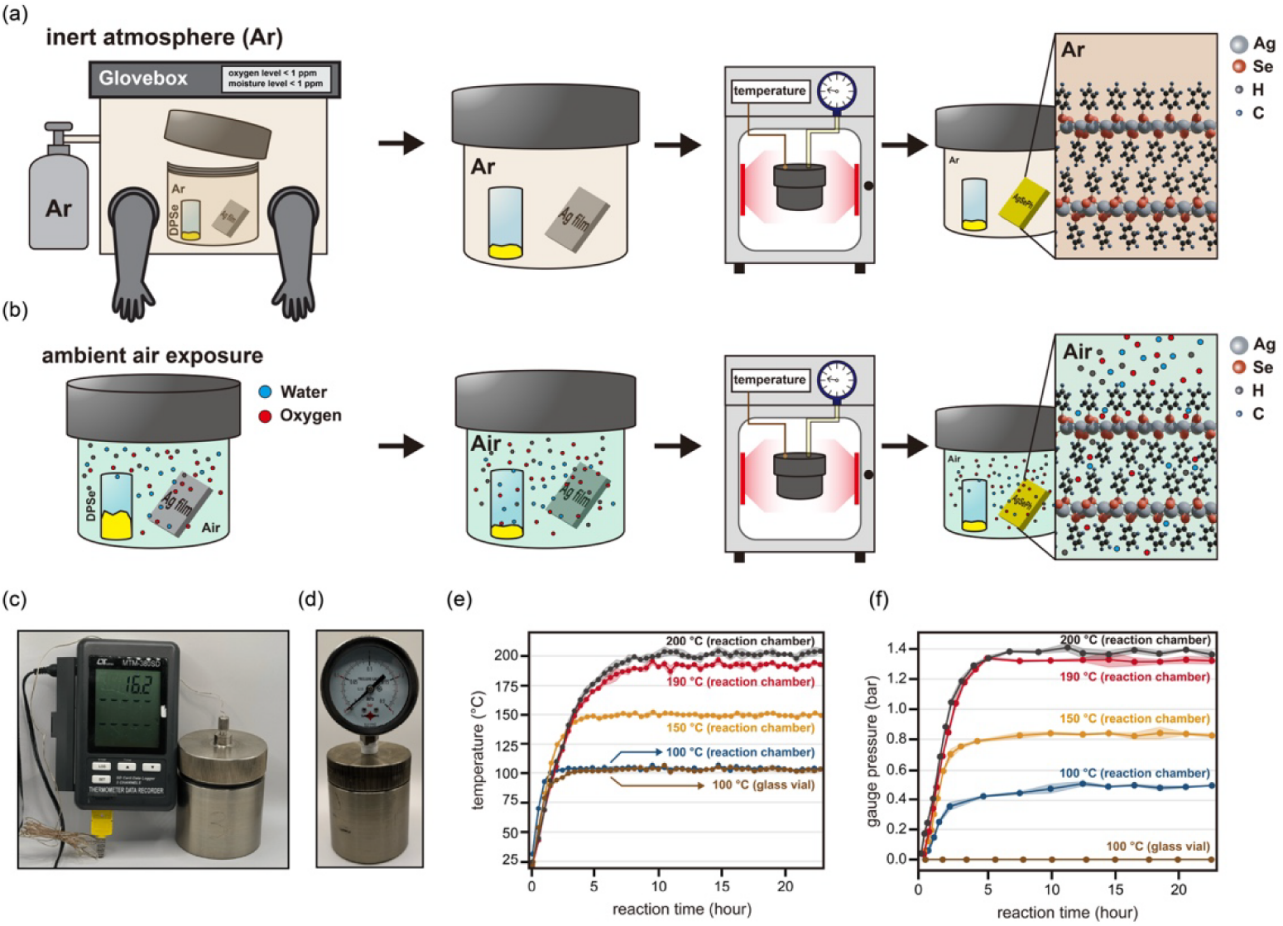
Overview of the custom-designed high-pressure inert environment reaction system. (a) Schematic of sample preparation and reaction setup within the argon-filled glovebox, ensuring an inert environment. (b) Comparative schematic of sample preparation in an air-exposed environment that is susceptible to contamination. Photographs of the experimental setup that show (c) the thermocouple and (d) the Bourdon-type pressure gauge for internal temperature and pressure monitoring. Plots of (e) the internal temperature and (f) corresponding internal pressure controllability within the sealed reaction chamber at various operating temperatures (100, 150, 190, and 200 °C), shown as averages of three independent measurements with shaded regions indicating the standard deviation.

A key feature of our sealed chamber is the ability to precisely regulate internal pressure through temperature modulation. The internal temperature and pressure were monitored using an integrated thermocouple and a Bourdon-type pressure gauge, respectively (Figure 1c, d). As shown in Figure 1e and f, the internal pressure in the chamber monotonically increased with increasing temperature (tested at temperatures of 100, 150, 190, and 200 °C), confirming that the reaction pressure can be effectively controlled by adjusting the system temperature. This precise pressure control, coupled with the maintenance of a high-purity (99.999%) argon atmosphere, effectively eliminates external interfering factors. Such controlled conditions are crucial for achieving reproducible synthesis of uniform, high-quality MOC films, irrespective of ambient laboratory conditions.^8,20^

To highlight the advantages of our custom-designed system, we conducted a comparative experiment using a conventional glass vial setup, which is a common configuration in previously reported solvent-assisted tarnishing methods (refer to Figure S2 for the setup). When heated to 100 °C, the internal pressure in the glass vial remained negligible (∼0 bar) even after thermal equilibrium was achieved (Figure 1e–f). This clearly demonstrates that standard glass vials lack the requisite sealing capability to generate or maintain elevated pressures under high-temperature conditions, resulting in no correlation between the external temperature and internal pressure. Consequently, such glass vial systems are unsuitable for creating high-pressure environments essential for optimizing MOC synthesis. The implications of solvent presence and atmospheric conditions are examined in greater detail in Section 2.6.

### 2.2 Influences of Temperature and Vapor Pressure on Mithrene Formation Kinetics and Film Morphology

The reaction kinetics of mithrene formation were systematically investigated as a function of the temperature and the corresponding internal vapor pressure generated in the sealed chamber. Figure 2a presents optical microscope images that capture the time-dependent morphological evolution of mithrene films synthesized under solvent-free conditions at 100 °C (∼0.5 bar), 150 °C (∼0.8 bar), and 190 °C (∼1.4 bar). These images clearly show three distinct reaction regimes: underreacted, optimally reacted, and overreacted regimes. Underreacted samples, characterized by partially unconverted silver regions indicative of incomplete reactions, were observed in samples reacted 24 h at 100 °C, 12 h at 150 °C, and 3–6 h at 190 °C. In stark contrast, optimally reacted films exhibited a uniform, bright yellow appearance, consistent with prior reports of fully formed mithrene, which was achieved within 36–48 h at 100 °C and 18–24 h at 150 °C, while a significant reduction of 12 h was achieved at 190 °C.^11,32^ Proceeding beyond this optimal point, the films entered an overreacted state, thus losing their characteristic yellow color and developing a dark brown color indicative of degradation. Such overreaction occurred within 60–72 h at 100 °C, 36–48 h at 150 °C, and 18–24 h at 190 °C. Notably, even at the lowest temperature of 100 °C, the optimal reaction window (36–48 h) in our system was considerably smaller than the >72 h typically needed in previously reported tarnishing methods using glass vials.^8,20^ Increasing the temperature greatly accelerated the reaction kinetics, thereby reducing the optimal window to approximately 50% (18–24 h at 150 °C) and 25% (12 h at 190 °C) of the time required at 100 °C. This acceleration was accompanied by a progressive narrowing of the optimal processing window.

**Figure 2:**
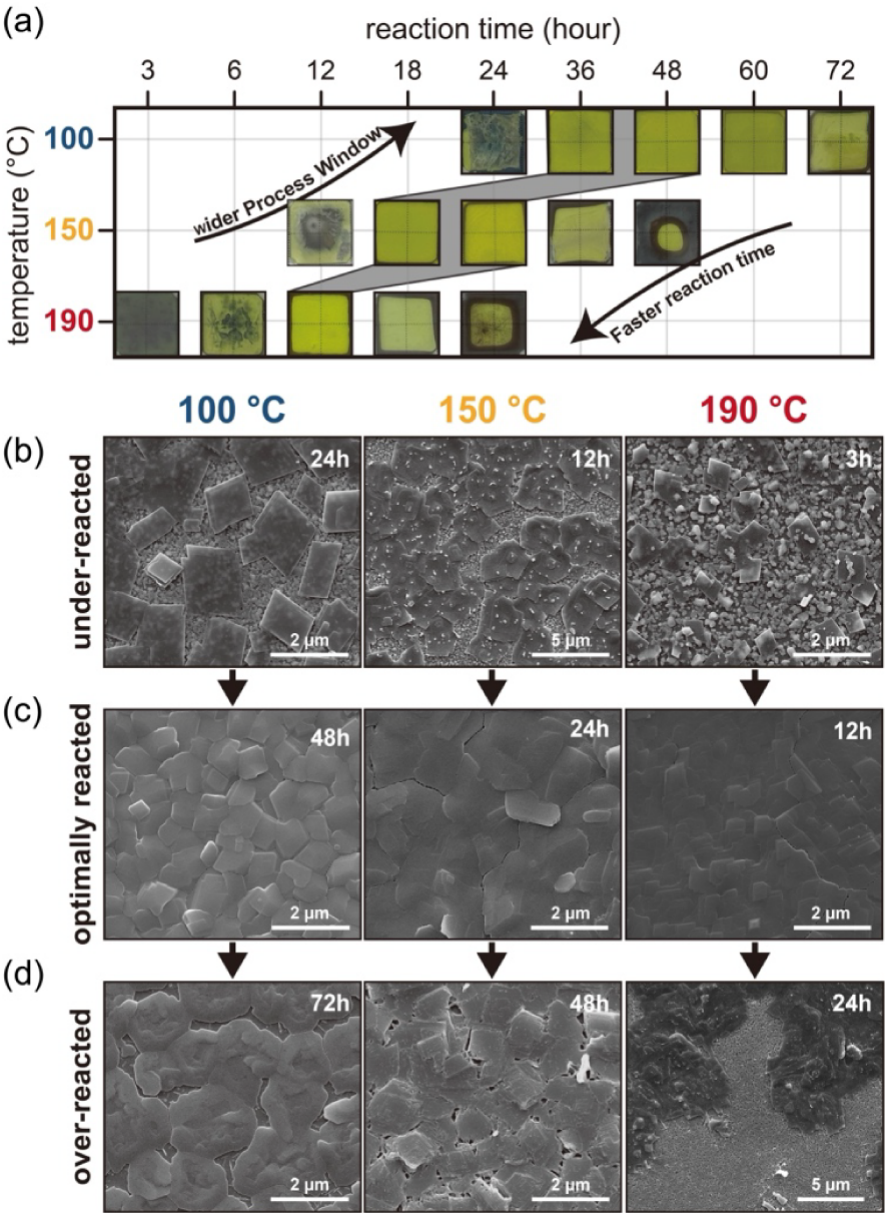
Evolution of mithrene films synthesized under solvent-free conditions at various temperatures and reaction times. (a) Optical images illustrating the visual progression from underreacted, through optimally reacted, to overreacted phases at temperatures of 100, 150, and 190 °C. Corresponding representative SEM images of the films at each temperature, showing the morphologies of the materials in the (b) underreacted, (c) optimally reacted, and (d) overreacted states.

The morphological evolution corresponding to these reaction stages was elucidated via scanning electron microscopy (SEM), with representative images shown in Figure 2b–d (additional time-dependent morphologies are provided in Figure S3). In the underreacted regime (Figure 2b), the SEM images revealed that isolated microcrystals, several micrometers in size, emerged as nucleation sites on the silver surface, indicating the early stages of film formation prior to complete conversion. Under optimal reaction conditions (Figure 2c), these microcrystals coalesced to form continuous and relatively uniform films. A significant temperature-dependent morphological transition was observed for these optimally reacted films: at 100 °C, the surface comprised uneven, overlapped crystalline domains with notable roughness. At 150 °C, the films became partially flattened, exhibiting increased grain connectivity and fewer discontinuities. A remarkable improvement occurred at 190 °C, in which the films demonstrated a strikingly flat and homogeneous morphology devoid of common surface defects such as cracks or pinholes, indicating superior structural quality. This trend, i.e., enhanced surface smoothness and structural integrity at higher temperatures, correlated well with the accelerated reaction kinetics, which could facilitate more complete and compact film growth within shorter durations. Conversely, the SEM images of the overreacted films (Figure 2d) clearly revealed degradation, including tearing and fragmentation of the film surface (see Section 5 in Supporting Information for optimal time).

Prolonged exposure to high DPSe vapor pressures at elevated temperatures led to the disruption of the layered mithrene structure and the eventual formation of silver clusters. SEM images clearly show this trend across reaction stages (Figure S5a). The corresponding XPS Ag 3d_5/2_ spectra (Figure S5b) show that the pristine Ag film exhibits a metallic peak at ∼368.11 eV, which shifts positively to ∼368.35 eV at the optimal state, consistent with Ag–Se ionic bonding. With further reaction, from the overreacted to fully degraded state, the peak reverts back to the metallic position, confirming ligand loss and the reaggregation of metallic Ag clusters. Raman spectra (Figure S5c) further support this interpretation: lattice modes of [AgSePh]_∞_ associated with the inorganic planes (<200 cm^–1^)^33^ are initially broad and weak in the under-reacted state, sharpen and become well resolved at the optimal state, then broaden again as the films enter the overreacted regime, and finally vanish completely in the fully degraded state. This systematic evolution of the lattice modes corroborates the collapse of the [AgSePh]_∞_ network.

Further investigation into higher-temperature regimes revealed that a temperature of 200 °C is detrimental to mithrene film formation, primarily because of thermal degradation, which is consistent with previous studies.^34,35^ In situ grazing incidence wide-angle X-ray scattering (GIWAXS) measurements (Figure S6a) provided direct evidence of this rapid structural collapse: characteristic diffraction peaks of the layered 2D structure, namely, (002), (004), and (006), began to diminish within approximately 1–2 min at 200 °C and completely disappeared after approximately 15 min. Having identified this rapid collapse, we attempted direct synthesis at 200 °C; however, no optimal condition could be achieved at this temperature. Instead, complementary optical images of samples, XPS analyses (Figure 6b–e) confirmed the absence of the bright yellow phase and the ideal Ag:Se stoichiometry, reinforcing that thermal decomposition dominates over crystal growth under these harsh conditions.

### 2.3 GIWAXS Elucidation of Structural Evolution Modulated by Reaction Kinetics

To better understand the structural evolution of mithrene films as a function of the reaction kinetics, we employed GIWAXS. Figure 3a–c show the 2D GIWAXS patterns for the films synthesized under underreacted, optimally reacted, and overreacted conditions at various temperatures. In defining these states, we note that the reaction times included in Figure 3 (100 °C: 24, 48, and 72 h; 150 °C: 12, 24, and 48 h; 190 °C: 3, 12, and 24 h) were chosen to represent the under-, optimal-, and over-reaction windows established earlier from PL and morphological analysis (Figure 2 and Figure S4). In the underreacted state (Figure 3a), all samples exhibited a diffuse Debye–Scherrer ring near *q* ≈ 2.6 Å^–1^, a characteristic signature of incomplete conversion attributable to residual silver, which is consistent with previous reports.^20^

**Figure 3:**
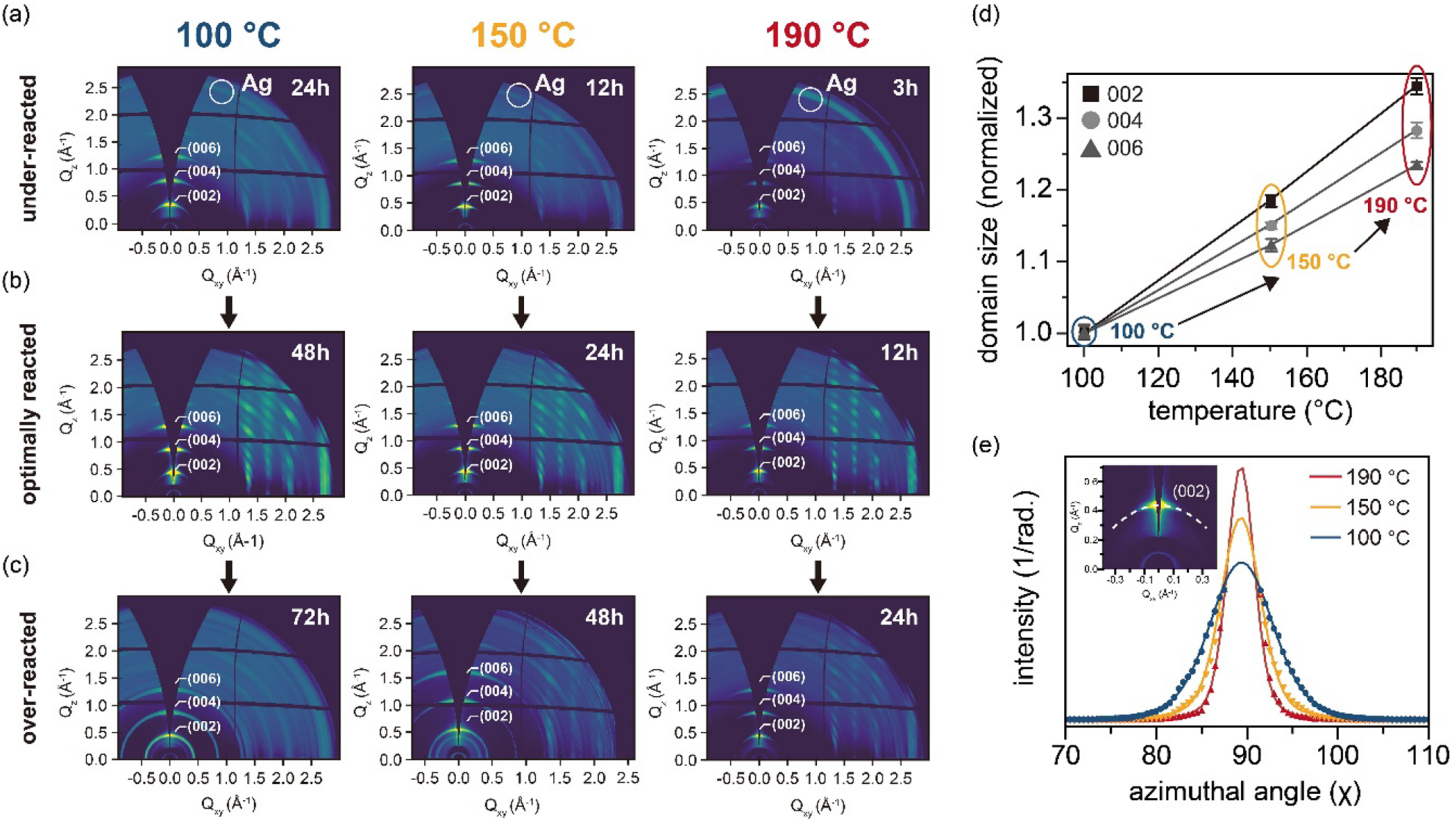
Structural characterization of mithrene films via GIWAXS across different reaction stages and synthesis temperatures. (a–c) Two-dimensional GIWAXS patterns of mithrene films representing (a) underreacted, (b) optimally reacted, and (c) overreacted states at each synthesis temperature. (d) Out-of-plane domain sizes, estimated from the (002), (004), and (006) diffraction peaks on the basis of the Scherrer equation, plotted as a function of the synthesis temperature for optimally reacted films, with fitting error included as error bars. (e) Area-normalized azimuthal orientation distribution (OD) profiles of the (002) reflection (inset) for optimally reacted films synthesized at 100, 150, and 190 °C. Experimental data points (markers) were extracted along the azimuthal direction in reciprocal q-space, with solid lines representing Voigt function fits, illustrating changes in the preferred orientation.

Conversely, films prepared under optimal reaction conditions (Figure 3b) exhibited pronounced, well-defined diffraction spots, indicative of a high degree of crystalline ordering. This ordering was observed not only along the characteristic out-of-plane (00*l*) direction but also along diagonal orientations such as (01*l*), (11*l*), (02*l*), and (20*l*), for which the detailed peak indexing is provided in Figure S8. To elucidate the specific crystal structure of optimally reacted film, we performed GIWAXS simulations on the basis of previously reported *P*2_1_/*c* and *C*2/*c* space groups.^21,36,37^ The experimental patterns conformed more closely with the *P*2_1_/*c* structure, suggesting that the mithrene films preferentially crystallize in this space group (see Section 8 of the Supporting Information for details).

Quantitative analysis of the GIWAXS patterns of the optimally reacted samples provided further insights into their structural quality. The out-of-plane domain sizes (γ) were estimated from the (002), (004), and (006) reflections (fitting results in Figure S9) via the Scherrer equation, i.e., $\gamma =\frac{K\lambda }{\beta \cos\left(\theta \right)}$ , where *K* is the shape factor (*K* = 0.94 for roughly spherical domains),^38,39^ λ is the X-ray wavelength, β is the full width at half-maximum (fwhm) of the diffraction peak, and θ is the Bragg angle.^40–42^ As shown in Figure 3d, the calculated domain size systematically increased with increasing synthesis temperature, peaking at 190 °C. This trend demonstrates that higher reaction temperatures, and thus optimized kinetics, facilitate the formation of more extended long-range ordering within the layered 2D mithrene lattice.

The in-plane crystal orientation of these optimally reacted films was assessed by analyzing the azimuthal orientation distribution (OD)^43–46^ of the (002) reflection (Figure 3e). The sharpness of the OD profiles progressively increased as the synthesis temperature increased to 190 °C, indicating an improved preferred orientation of the layered-structure domains along the out-of-plane direction. This sharpening corresponded to a reduction in mosaicity (i.e., a narrower distribution of crystallite orientations), which corroborates the SEM observations of flatter and more uniform film surfaces at higher temperatures.^47,48^ These structural enhancements, particularly the reduction in lattice strain and the improvement in orientational order, could strongly enhance charge transport properties, making the films promising candidates for advanced optoelectronic applications.

Finally, in the overreacted regime (Figure 3c), the GIWAXS patterns revealed notable broadening of the diffraction peaks, particularly along the azimuthal direction. This broadening reflects increased mosaic spread and partial disruption of the layered crystalline structure. To illustrate this degradation, azimuthal OD profiles of the (002) peak for all three reaction phases at each temperature are shown in Figure S10. These profiles clearly demonstrate that the sharp OD profile observed under optimal conditions deteriorates upon overreaction, providing robust structural evidence that complements the SEM-based morphological analysis.

### 2.4 XPS Analysis of Stoichiometric Evolution and Chemical Uniformity Modulated by Reaction Kinetics

To investigate the influences of the reaction kinetics on the chemical structure and stoichiometry of the mithrene films, we performed XPS measurements. Figure 4a–c shows the C 1s, Ag 3d, and Se 3p core-level spectra of the films synthesized at temperatures of 100, 150, and 190 °C across various reaction times. Mithrene ideally comprises a 2D AgSe framework with phenyl groups, corresponding to an atomic ratio of Ag:Se:C_C–Se_ = 1:1:1, and within the phenylselenolate ligand, the C_C–C_:C_C–Se_ ratio is 5:1 (Figure 4d). The C 1s spectra (Figure 4a–c) were deconvoluted into components representing C–C bonds (from the phenyl ring, orange, centered at ∼284.6 eV) and C–Se bonds (blue, centered at ∼286.0 eV), which is consistent with the 1:5 ratio of carbon atoms directly bonded to selenium versus other carbon atoms in the phenylselenolate structure. The results of quantitative analysis of the deconvoluted C 1s, Se 3p, and Ag 3d peak intensities are shown in Figure 4f and g, while the detailed absolute atomic percentages corresponding to each spectrum are provided in Table S2. The Se:C_C–Se_ atomic ratio remained largely constant across all the spectra (Figure 4f), indicating that the relative selenium content tied to the phenyl group does not significantly change with reaction time or temperature.

**Figure 4:**
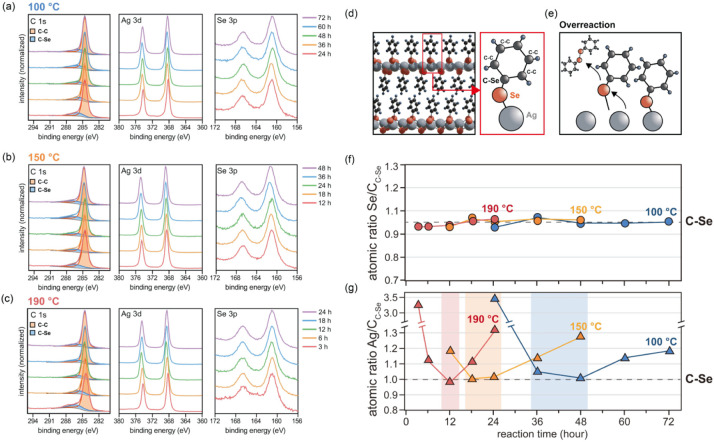
XPS analysis of mithrene films revealing chemical bonding and stoichiometry as a function of the reaction conditions. Core-level spectra (C 1s, Ag 3d, and Se 3p) of the mithrene films synthesized at (a) 100 °C, (b) 150 °C, and (c) 190 °C. (d) Schematic of the ideal mithrene chemical structure, illustrating the atomic ratios C_C–C_:C_C–Se_ = 5:1 and Ag:Se:C_C–Se_ = 1:1:1. (e) Conceptual depiction of phenylselenolate ligand detachment from Ag atoms during overreaction. Relative atomic ratios of (f) Se to C_C–Se_ and (g) Ag to C_C–Se_ as a function of the reaction time at each temperature, derived from XPS peak areas, considering atomic sensitivity factors.

In contrast, the Ag:C_C–Se_ ratio (Figure 4g) deviated from the ideal 1:1 stoichiometric composition under both under- and overreacted conditions. This ideal 1:1 ratio was achieved only at the optimal reaction time for each temperature. Furthermore, no notable binding energy shift was observed in the Se 3p core-level spectra across all conditions (exemplified in Figure 4c for the 190 °C samples), suggesting that selenium does not undergo chemical conversion into other phases during the reaction. These observations support a mechanistic interpretation wherein phenylselenolate ligands become progressively coordinated with Ag atoms during the transition from underreacted to optimally reacted phases, thus forming the layered mithrene structure. Conversely, under overreacted conditions (conceptually shown in Figure 4e), these ligands likely detach, leaving behind silver atoms that subsequently aggregate into cluster-like domains, a phenomenon corroborated by the SEM imaging (Figure S5a). Notably, Figure 4g also reveals that at lower temperatures, the Ag:C_C–Se_ ratio approaches the ideal 1:1 value more gradually. This slower convergence reflects the reduced reaction kinetics at lower temperatures and results in a broader optimal processing window (36–48 h at 100 °C, 18–24 h at 150 °C), in contrast to the sharply confined window at 190 °C (12 h).

To assess the chemical uniformity of the films along the vertical direction, we conducted XPS depth profiling via Ar sputtering on an optimally reacted mithrene film synthesized at a temperature of 190 °C (Figure S11). Crucially, no binding energy shift was observed in the Ag 3d_5/2_ core-level spectrum relative to its pristine metallic peak (centered at ∼368.0 eV) throughout the sputtering process until the selenium signal completely disappeared (Figure S11b) and the underlying indium tin oxide (ITO) substrate was exposed (Figure S11c). This consistent Ag 3d_5/2_ peak position confirms that the silver within the mithrene film maintains its chemical state and that the chemical integrity of the film is preserved throughout its depth. This finding substantiates the high quality and chemical homogeneity of the mithrene films synthesized via our solvent-free approach.

### 2.5 Influence of Reaction Kinetics on Optical Properties: Excitonic Behavior and Photoluminescence

The impact of the variation in reaction kinetics on the optical properties of the synthesized mithrene films was investigated through UV–vis absorption and photoluminescence (PL) spectroscopy. Figure 5a–c shows the time-dependent evolution of the optical spectra for the films prepared at 100, 150, and 190 °C. The overall shapes of both the absorption and PL spectra were consistent across all the samples and conformed well with previously reported data.^6,9,10,33^ To obtain greater insight into the individual excitonic transitions within the absorption spectra, we performed a second derivative analysis (Figure S12a). This analysis revealed three distinct excitonic peaks: X_1_ at ∼464 nm, X_2_ at ∼455 nm, and X_3_ at ∼434 nm. Such transitions exhibit high spatial anisotropy, with the X_1_ and X_3_ excitons associated with the transition dipole moment along the [010] direction and the X_2_ exciton along the orthogonal [100] direction.^9^ The absorbance intensities of the X_2_ and X_3_ features extracted from the spectra are shown in Figure 5d and e, respectively. In both instances, the highest absorbance intensities were consistently observed for films prepared under the optimal reaction conditions (indicated by red, orange, and blue boxes for 100, 150, and 190 °C, respectively). Notably, samples reacted at 190 °C demonstrated the highest overall absorption compared with those prepared at lower temperatures, a finding that is strongly correlated with the superior crystallinity observed via GIWAXS analysis (Figure 3b). Similarly, the PL intensity trends, plotted as a function of the reaction time for each temperature in Figure 5f, mirrored the absorption behavior. The PL intensity peaked under optimal conditions, with higher emission indicating higher crystalline quality.

**Figure 5:**
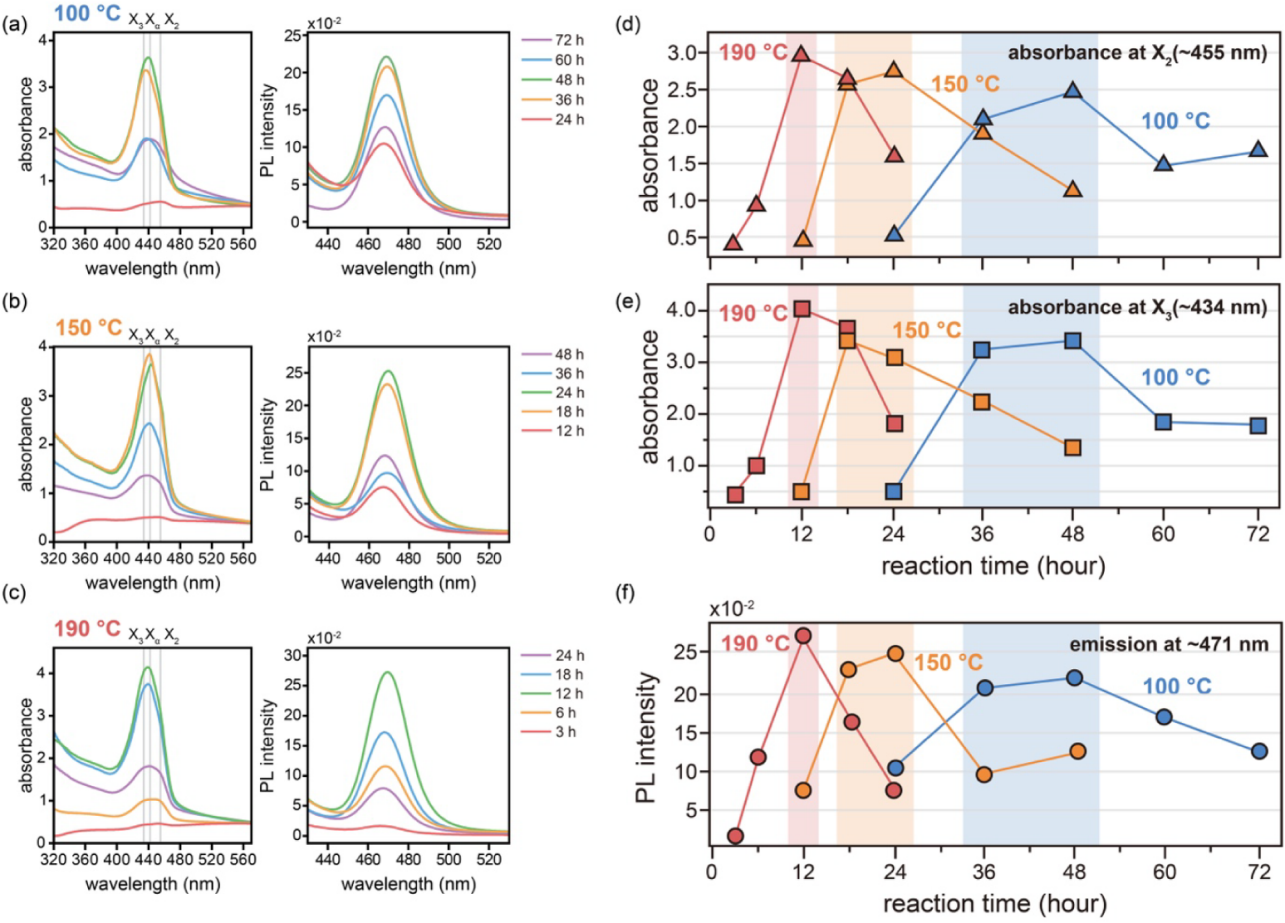
Optical properties of mithrene films as a function of the synthesis temperature and reaction time. Time-dependent evolution of UV–vis absorption and photoluminescence (PL) spectra for mithrene films synthesized at temperatures of (a) 100 °C, (b) 150 °C, and (c) 190 °C, showing changes during reaction progression. (d, e) Absorbance evolution of the X_2_ (∼455 nm) and X_3_ (∼434 nm) excitonic features as a function of the reaction time at each temperature. (f) Corresponding time-dependent PL intensity at ∼471 nm for films synthesized at each temperature, overlaid for direct comparison, highlighting the impact of reaction conditions on the emission strength.

Intriguingly, the second derivative analysis of the absorption spectra consistently revealed a previously unreported excitonic feature, designated X_α_, which emerged at ∼446 nm and occurred between the X_2_ and X_3_ transitions. This X_α_ peak was most prominent in films synthesized under the optimal reaction conditions at each temperature, whereas the peak intensity decreased significantly in both the underreacted and overreacted samples. Although the precise origin of this X_α_ feature remains to be elucidated, the association of its emergence with optimal conditions suggests that it may represent an excitonic transition enabled or enhanced by the high degree of structural coherence and crystallinity achieved in these mithrene films. Further investigations are warranted to better understand the underlying mechanism of this new excitonic state.

### 2.6 Superiority of Solvent-Free, Inert Atmosphere Synthesis: A Comparative Study of Environmental Effects

Our preceding experimental findings (Sections 2.2–2.5), which were achieved via the solvent-free, high-pressure inert system, clearly indicate that solvent assistance is not intrinsically necessary for the production of high-quality mithrene films. These results highlight that the crucial parameters are the reaction temperature and internal vapor pressure, both of which are precisely controllable within our custom-designed sealed reaction chamber. This capability contrasts sharply with earlier studies involving the use of glass vials, which, as was verified, possess insufficient sealing capacity to increase the internal pressure via temperature modulation alone (an internal pressure of ∼0 bar at 100 °C, Figure S14). This limitation likely accounts for previous discrepancies and the perceived necessity of assistant solvents in those systems.^8,20^

To investigate the individual effects of atmospheric conditions and solvents under controlled high-pressure conditions, we synthesized mithrene films in our reaction chamber in five distinct environments, all of which were maintained under identical temperature (100 °C) and internal pressure (∼0.5 bar, Figure S15) conditions: (i) Ar atmosphere (solvent-free), (ii) air atmosphere (solvent-free, ∼40% relative humidity at room temperature), (iii) air + DI water, (iv) air + DMSO, and (v) air + PrNH_2_. While the SEM images revealed similar lamellar microstructures with rectangular domains across all the samples (Figure 6a), their optical images (insets, Figure 6a) clearly showed macroscopic differences. The films synthesized in Ar (i) exhibited a uniform and bright yellow appearance, whereas those prepared in air (ii) or with air + assistant solvents (iii–v) demonstrated localized dark spots, indicating nonuniformity.

**Figure 6:**
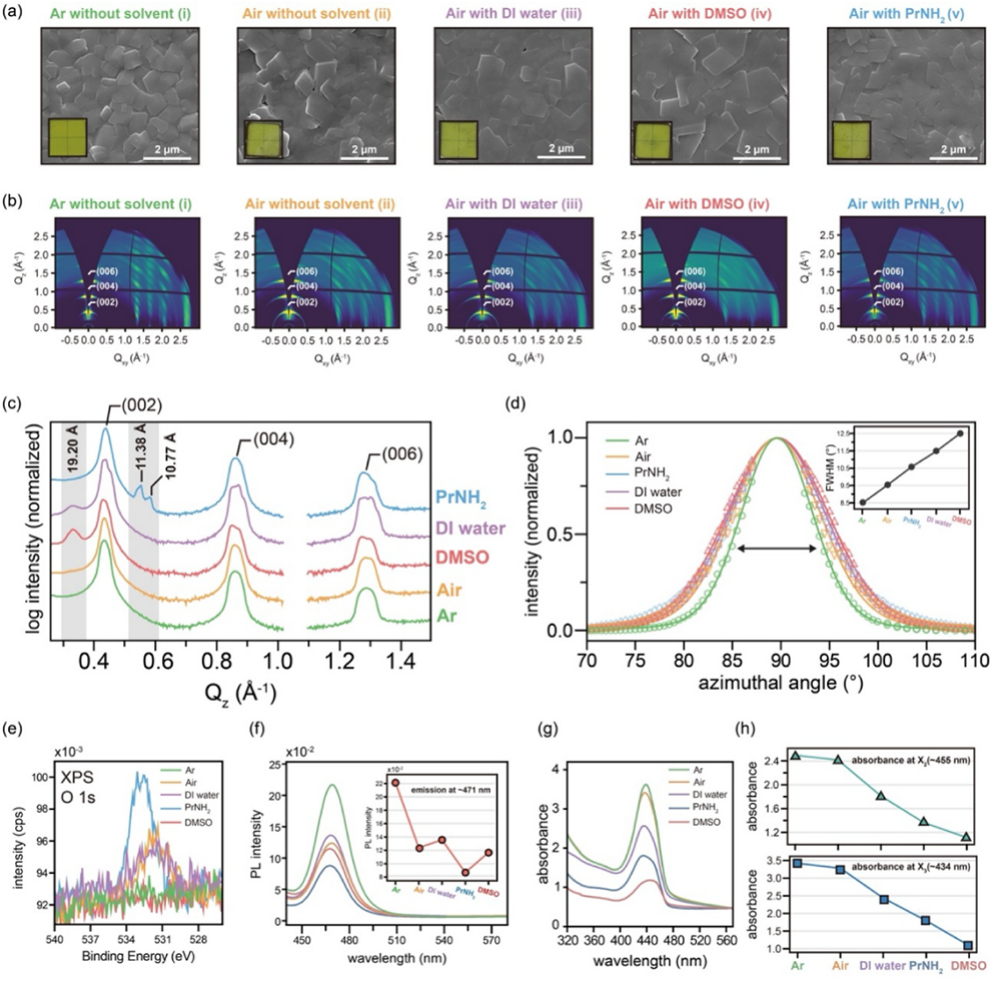
Comparative analysis of mithrene films synthesized in various chemical environments within a controlled high-pressure reaction chamber. The conditions are as follows: (i) Ar (solvent-free), (ii) air (solvent-free), (iii) air + deionized (DI) water, (iv) air + DMSO, and (v) air + PrNH_2_. (a) SEM images with corresponding optical image insets. (b) Two-dimensional GIWAXS patterns. (c) Extracted out-of-plane 1D GIWAXS profiles. (d) Normalized azimuthal OD profiles for the (002) reflection; the markers indicate experimental data, the solid lines indicate Voigt fits, and the inset shows the fwhm values. (e) XPS O 1s core-level spectra indicating surface oxidation. (f) PL spectra with an inset of PL intensities at ∼471 nm. (g) UV–vis absorption spectra. (h) Quantitative comparison of X_2_ (∼455 nm) and X_3_ (∼434 nm) excitonic absorption intensities.

GIWAXS analysis elucidated the structural differences ([Fig fig6]b–d). The film synthesized under an Ar atmosphere (i) exhibited sharp diffraction spots, particularly along diagonal orientations, indicating a high degree of crystalline alignment (Figure 6b). In contrast, films prepared under air (ii) or air with solvents (iii–v) exhibited broader and weaker diffraction patterns, indicative of increased azimuthal disorder. The 1D out-of-plane GIWAXS profiles (Figure 6c) confirmed regular lamellar stacking ((002), (004), and (006) reflections) for the samples prepared under Ar (i) and air (ii) conditions. However, the films synthesized in the presence of solvents (air + DI water (iii), air + DMSO (iv), air + PrNH_2_ (v)) demonstrated additional peaks (e.g., at *d* = 19.2 Å for iii and iv and new peaks at 11.38 and 10.77 Å for (v) not corresponding to known mithrene interlayer spacings (confirmed by GIWAXS simulation, Figure S8a), suggesting the formation of structural defects, abnormal phases or intercalation resulting from air contained impurities such as H_2_O, O_2_, CO_*x*_ or CH_*x*_. Similarly, the normalized azimuthal OD profiles for the (002) reflection (Figure 6d) were the sharpest for the Ar (i) sample (fwhm ≈ 8.5°), followed by the air (ii) sample, whereas all the solvent-containing samples (iii–v) demonstrated significantly broader distributions, indicating the presence of more randomly oriented microcrystals and the disruption of mosaic order by solvents.

XPS analysis provided insights into the chemical state. The O 1s core-level spectra (Figure 6e) revealed surface oxidation in the films exposed to air (ii), air + DI water (iii) and those synthesized with PrNH_2_ (v), likely due to ambient moisture and oxygen. Conversely, the Ar (i) sample indicated no O 1s peak, demonstrating the effectiveness of the inert atmosphere in preventing contamination. This finding was further supported by Ag:Se atomic ratio analysis (Figure S16), in which only the Ar (i) sample exhibited the ideal stoichiometry, whereas the air-exposed samples showed increasing deviations.

The optical properties of these films also varied significantly with synthesis environment (Figure 6f–h). The film synthesized in Ar (i) exhibited the highest PL intensity and the most pronounced excitonic absorption features. In contrast, the films synthesized in air (ii) or with air and solvents (iii–v) showed a notable reduction in the overall optical performance, including suppressed excitonic features (shown in the insets of Figure 6f and h), which is likely attributable to the increased structural imperfections such as structural defects, chemical impurities, grain boundaries, or residual strain.^49,50^

In summary, these comparative results unequivocally demonstrate that a fully sealed, inert, and solvent-free environment, as achieved in our custom reaction chamber, is highly beneficial for synthesizing high-quality mithrene films. This approach promotes superior crystallinity, prevents oxidation and contamination, and enables the reproducible fabrication of structurally ordered and optically superior mithrene films, irrespective of external atmospheric variables.

## 3 Conclusion

In conclusion, a novel solvent-free synthesis methodology for producing high-quality mithrene thin films in a custom-designed high-pressure inert environment was successfully introduced and validated. Through precise temperature-mediated control of the internal vapor pressure, we achieved systematic optimization of the reaction kinetics, enabling the formation of continuous films with significantly enhanced crystallinity, larger domain sizes, improved crystal orientation, and excellent chemical stoichiometry and uniformity, all without the need for solvent assistance. Notably, the films synthesized at higher temperatures under these optimized conditions exhibited superior structural characteristics that correlated directly with enhanced excitonic absorption and greater photoluminescence, alongside the observation of a previously unreported excitonic feature (X_α_), which possibly relates to high structural coherence. These comprehensive findings clearly demonstrate that under sufficiently controlled high-pressure and high-temperature conditions, solvent participation is not a prerequisite for mithrene synthesis, challenging the notion of solvents as chemically essential components in such processes. Further, the solvent-free approach minimizes chemical handling and waste, thereby providing practical advantages in safety, reproducibility, and cost over solvent-assisted routes. Our approach therefore offers a robust and reproducible pathway for advancing the synthesis of mithrene thin films and other layered MOCs, significantly enhancing their potential for integration into next-generation optoelectronic devices such as photodetectors, opto-neuromorphic devices, and light-emitting diodes. Furthermore, the fundamental insights into the reaction kinetics established herein could be transferred to the synthesis of a wider palette of MOC thin films, including AuSePh and CuSePh, thereby paving the way for an expanded family of functional hybrid quantum well materials.

## 4 Experimental Section

### 4.1 Sample Preparation

Indium tin oxide (ITO) substrates were sequentially cleaned by ultrasonication in deionized (DI) water, detergent, acetone, and ethanol, each for 15 min, followed by drying under nitrogen flow. To remove residual organic contaminants, the substrate was treated with ultraviolet (UV)-ozone at 100 °C for 15 min. A 15 nm-thick silver film was deposited onto the cleaned ITO substrates via electron beam (e-beam) evaporation using a ULVAC e-beam evaporator with 99.99% pure Ag source. The deposition was performed under a pressure of 4.1 × 10^–4^ Pa and at a rate of 0.5 Å·s^– 1^. Prior to each synthesis, the stainless-steel reaction chamber was thoroughly purged by seven consecutive cycles of Ar (99.999% purity refill and evacuation to −0.1 bar, with the lid kept open, to remove residual oxygen and moisture inside of the chamber. The chamber was then transferred into a catalyst-based Ar glovebox (O_2_ < 0.1 ppm, H_2_O < 0.1 ppm). Inside the glovebox, 35 mg of diphenyl diselenide (DPSe, 97.0%, Tokyo Chemical Industry Co., Ltd.) powder and the Ag-coated ITO substrate were placed into the reaction chamber. The lid was then tightly sealed against the Viton O-ring (Korea New Technology Co.) interface to ensure complete isolation. The sealed chamber was placed inside a laboratory oven (Lab Commerce Co.) preheated to the target reaction temperature (100, 150, or 190 °C). For pressure safety reason, the chamber was only opened once it had cooled to room temperature. At this point, the DPSe vapor had fully recondensed into the solid phase, causing the internal pressure to drop close to ambient, which allowed the chamber to be safely opened without sudden gas release and impact. After each run, selenium-containing byproducts deposited on the chamber wall and lid were removed by annealing the components at 300 °C for 1 h inside a fume hood, followed by sonication in isopropanol (IPA).

### 4.2 Pressure Sensor, Leak Testing, and Safety Assurance of the Reaction Chamber

A Bourdon-type pressure gauge (accuracy ± 1.5%, SAMSUNG INSTRUMENT Co.) was calibrated in accordance with the Korean national standard (KS B 5305) using incremental pressurization and depressurization cycles, confirming that the readings were reproducible and within the certified tolerance. Before synthesizing the sample, the prepared reaction chamber was subjected to a leak test under conditions harsher than the synthesis conditions. The chamber was heated to 230 °C and maintained for 72 h while the internal pressure was continuously monitored. The pressure increased to ∼2.0 bar within 8 h, and subsequently remained stable at ∼2.0 bar for the next 60 h. This confirms that the chamber can be safely operated for extended durations under high-temperature and high-pressure conditions. High pressure can potentially cause safety issues with the chamber. For example, glass vials have occasionally fractured at high temperatures above 170 °C. Therefore, we conducted our experiments within the standard safety ratings of the stainless-steel chamber and the Viton O-ring. The stainless-steel body used in this study is generally known to be safe at pressures exceeding several hundred bar, and the Viton O-ring is safe up to ∼250 °C and ∼10 bar under typical sealing conditions. Thus, our experiments, which were conducted below 10 bar and 250 °C, were within the safe operating range.

### 4.3 SEM Measurement

Surface morphologies of the mithrene films were characterized by field-emission scanning electron microscopy (FE-SEM; Inspect F50, FEI) using a secondary electron (SE) detector operated at an acceleration voltage of 10 kV under ultrahigh vacuum (UHV) conditions. Prior to imaging, the samples were coated with a thin platinum (Pt) layer using an ion sputter coater (Hitachi E-1045) at 15 mA for 45 s under medium vacuum to minimize charging effects.

### 4.4 GIWAXS Measurement

Grazing incidence wide-angle X-ray scattering (GIWAXS) measurements were performed at the 3C SAXS beamline of the Pohang Accelerator Laboratory (PAL), Pohang (Republic of Korea). The photon energy for the experiment was 11.3 keV (λ = 1.097 Å). The beam size used in GIWAXS was ∼350 × 100 μm^2^, and the intrinsic contribution is estimated to be Δ*q* ≈ 0.01 × 0.0035 Å^–1^, which is far smaller than the broadening caused by sample-intrinsic broadening. The samples were placed 213 mm upstream from the detector position. For the grazing-incidence measurement, the sample tilt angle was 0.05°. To collect the scattered image, an Eiger X4M (Dectris) detector with 75 × 75 μm^2^ pixel size was used.

### 4.5 X-ray Photoelectron Spectroscopy (XPS) Measurement

XPS measurements were conducted using a ThermoFisher NEXSA system equipped with a monochromatic Al Kα source (1486.8 eV) under ultrahigh vacuum (UHV) conditions, and energy calibration was performed using the Au 4f_7/2_ core level of a freshly sputter-cleaned Au (111) surface, fixed at 84.0 eV. Spectra were acquired with a spot size of 100 μm, pass energy of 50 eV, dwell time of 25 ms, and step size of 0.05 eV, and a takeoff angle aligned with the sample surface normal. Depth profiling was performed using Ar^+^ sputtering with an ion energy of 2.0 keV and raster size of 1.00 mm. The sputtering rate was calibrated to 1.56 nm/s using a Ta_2_O_5_ reference, and each etch cycle consisted of a 20 s sputtering interval.

### 4.6 Optical Measurement

UV–vis absorption spectra were measured using a UV–vis spectrophotometer (V-730, JASCO) in transmission mode, with the film side facing the excitation beam. Baseline correction was performed using a cleaned ITO substrate. The measurement was carried out with a data interval of 0.5 nm, a spectral bandwidth of 1.0 nm, and a response time of 0.06 s. Photoluminescence (PL) spectra were measured using a JASCO FP-8500 fluorescence spectrophotometer. The excitation wavelength was set to 405.0 nm using a xenon (Xe) lamp as the light source. The scan was performed at a speed of 1000 nm/min with a data interval of 1 nm and a response time of 1 s. All measurements were conducted at room temperature under air atmosphere.

## Supplementary Material



## Notes

Comparison of reported mithrene synthesis methods and reaction conditions; geometrical layout and design of the custom stainless-steel reaction chamber; experimental setup for pressure calibration using a glass vial; morphological evolution of mithrene films at different synthesis temperatures; photoluminescence analysis for determining the optimal reaction window; morphological, chemical, and vibrational degradation of samples synthesized at 190 °C; thermal degradation and temporal evolution of mithrene films at 200 °C; GIWAXS measurements, structural simulation, and out-of-plane linecut analyses with fwhm fitting; XPS quantification, absolute atomic percentages, and depth profiling; UV–vis absorption and PL evolution analyses; temperature–pressure measurements under air and solvent environments; and characterization of mithrene films synthesized under N_2_ atmosphere (PDF)
