# Supplementary material for: Kinetics Control of Mithrene Formation in a High-Pressure Inert Environment: A Robust Solvent-Free Route to Superior-Quality Films
Source: ACS Appl Mater Interfaces. 2025 Nov 3;17(48):65956–66. doi: 10.1021/acsami.5c15192 (PMC12679529; doi:10.1021/acsami.5c15192)
Supplement: Supplementary file 1 [file am5c15192_si_001.pdf]

Supporting Information

Kinetics Control of Mithrene Formation in a High-Pressure Inert Environment: A Robust Solvent-Free Route to Superior-Quality Films

Seunghwan Kim[1, 2], Kitae Kim[1, 2], Aelim Ha[1, 3], Eunki Yoon[1, 3], Sooyeon Pak[1, 2], Eunjong Yoo[2], Ki Hoon Nam[4], Seung Min Kwak[4], Won Kook Choi[5], Young Yong Kim[6], Kyu Hyoung Lee[3], Yeonjin Yi[2, *], Soohyung Park[1, 7, *]

[1]Advanced Analysis and Data Center, Korea Institute of Science and Technology (KIST), Seoul 02792, Republic of Korea

[2]Department of Physics, Yonsei University, 50 Yonsei-ro, Seodaemun-gu, Seoul 03722, Republic of Korea

[3] Department of Materials Science and Engineering, Yonsei University, Seoul 03722, Republic of Korea

[4]Micro/Nano Fabrication Center, KIST, Seoul 02792, Republic of Korea

[5]Center for Opto-Electronic Materials and Devices, KIST, Seoul 02792, Republic of Korea

[6]Beamline division, Pohang Accelerator Laboratory, POSTECH, Pohang 37673, Republic of Korea

[7]Division of Nano & Information Technology, KIST School, University of Science and Technology (UST), Seoul 02792, Republic of Korea

*E-mail: yeonjin@yonsei.ac.kr, soohyung.park@kist.re.kr

TABLE OF CONTENTS

Section 1. Comparison of Reported Mithrene Sampling Methods......................................4

Section 2. Geometric Layout of the Reaction Chamber..5

Section 3. Glass Vial Setup for Pressure Measurement..6

Section 4. Morphological Evolution of Mithrene Films at Different Temperatures..........7

Section 5. Determination of Optimal Reaction Window from PL Analysis.......................8

Section 6. Morphological, Chemical, and Vibrational Evolution and Complete Degradation of Mithrene Films at 190 °C..10

Section 7. Thermal Degradation and Temporal Evolution of Mithrene Films at 200 °C..11

Section 8. GIWAXS Analysis and Structure Simulation of Mithrene Film.....................12

Section 9. Out-of-Plane GIWAXS Profiles and FWHM Analysis....................................15

Section 10. Reaction Temperature-Dependent Orientation Distribution of Solvent-Free Mithrene Films...16

Section 11. Absolute Atomic Percentages from XPS Quantification.................................17

Section 12. XPS Depth Profiling of Optimally Reacted Mithrene Film Synthesized at 190 °C..18

Section 13. Second-derivative Analysis of UV-vis Absorption Spectra............................19

Section 14. Temporal Evolution of Absorbance Trends at ~464 nm (X_1), ~446 nm (X_a)..20

Section 15. Temperature and pressure measurements in conventional glass vial setups under air (solvent-free) and air + solvent conditions at 100 °C......................21

Section 16. Thermal and Pressure Profiles in the Reaction Chamber under Air and Air + Solvent Conditions at 100 °C...22

Section 17. XPS Spectra of Samples Synthesized under Different Environments...........23

Section 18. Second-derivative Spectra Analysis and Absorbance Trends of Solvent-Free and Solvent-Assisted Samples...24

Section 19. Characterization of Mithrene Films Synthesized under N_2 Atmosphere......25

Section 20. Comparative Structural, Chemical, and Optical Properties under Different Reaction Conditions..27

Reference. 28

Section 1. Comparison of Reported Mithrene Sampling Methods

Methodology	Reaction Temperature	Use of solvent	Reaction chamber	Optimal reaction time	Journal Information
Tarnishing	100 °C, 150 °C, 190 °C	No Solvent	Stainless-steel Reaction Chamber	48 hours, 24 hours, 12 hours	This work
Tarnishing	100 °C	DI Water	Sealed vial	72 hours	ACS Nano 2022, 16, 12, 20318–20328[1]
Tarnishing	80 °C	DI Water	Sealed jar	72 hours	J. Am. Chem. Soc. 2018, 140, 42, 13892–13903[2]
Tarnishing	90 °C	Solvent-free	Sealed vial	30 minutes	J.Phys.Chem.C2020,124,22845–22852[3]
Tarnishing	100 °C	hexane, heptane, octane, decane, and more	Sealed vial	72 hours	ACS Nano2022,16,2054−2065[4]
Tarnishing	100 °C	ethanol	Sealed jar	48 hours	Dalton Trans., 2025,54, 12970-12978[5]
Biphasic	RT	-	Glass vial	72 hours ~ 120 hours	ACS Appl. Nano Mater. 2018, 1, 3498–3508[6]

Table S1 Summary of reported synthesis methods for mithrene (AgSePh) films and nanocrystals. The table compares reaction temperature, solvent usage, reaction chamber type, and the corresponding optimal reaction time.

Section 2. Geometric Layout of the Reaction Chamber

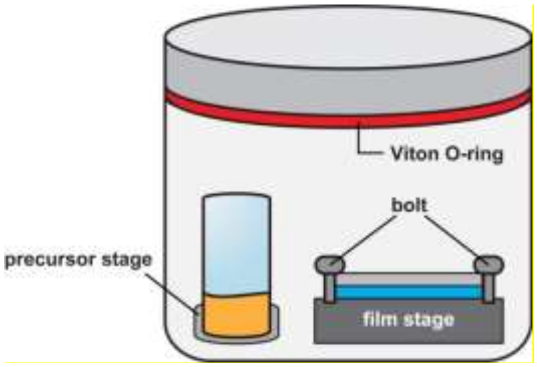

Figure S1 Schematic illustration of the custom-designed reaction chamber. The cylindrical body with a detachable threaded lid compresses against a Viton O-ring to ensure airtight sealing. The interior incorporates a precursor stage (left) and a film stage (right) mounted on the lid, providing stable placement and a well-defined spacing between the precursor source and the Ag substrate during solvent-free reactions.

The reaction chamber has a compact cylindrical geometry with a detachable threaded lid that compresses against a Viton O-ring to ensure airtight sealing. The interior incorporates dedicated stages that allow stable placement of both the precursor source and the Ag substrate. The lid also includes a central mounting area to hold the Ag film in a fixed position. This configuration maintains a well-defined spacing between precursor and substrate, while providing reproducible alignment for solvent-free reactions.

Section 3. Glass Vial Setup for Pressure Measurement

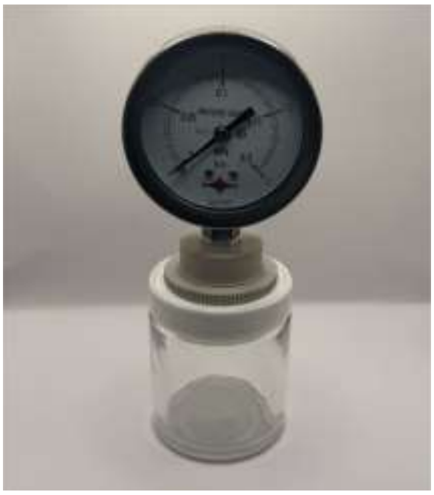

Figure S2 A photograph of the experimental setup for internal pressure measurement using conventional glass vial.

To evaluate the sealing performance of conventional glass vial systems, we conducted pressure measurements using the setup shown in Figure S2. The system consisted of a glass vial sealed with a polytetrafluorethylene (PTFE) insert glued onto a polypropylene (PP) cap. A Bourdon-type pressure gauge was mounted onto the PP cap.

Section 4. Morphological Evolution of Mithrene Films at Different Temperatures

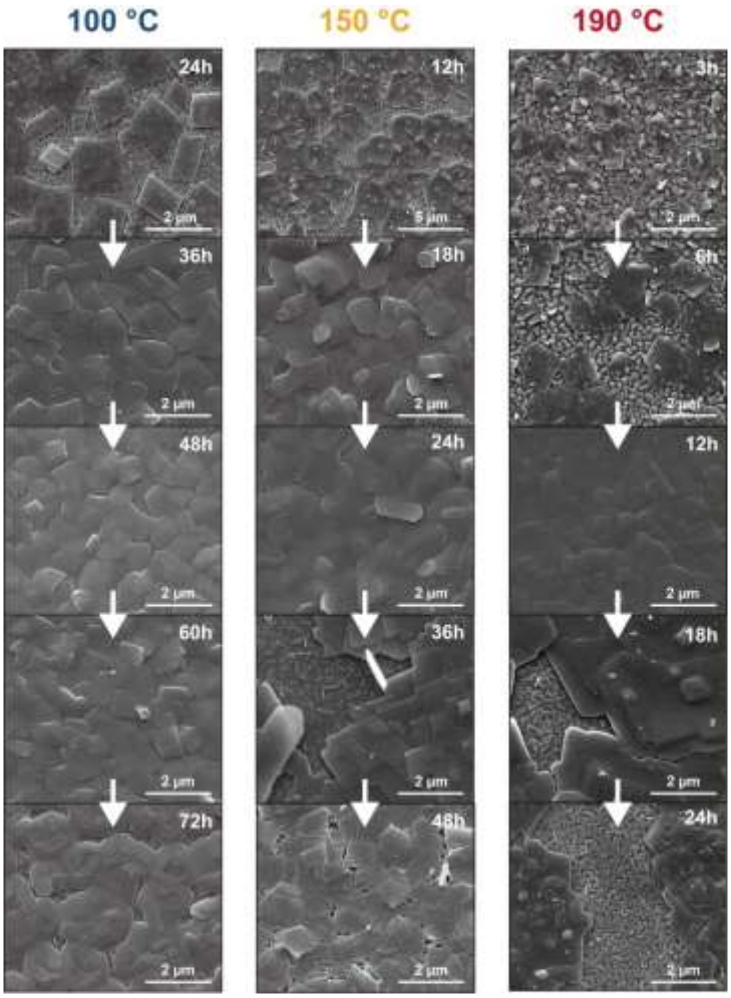

Figure S3 Comprehensive view of the morphological evolution of mithrene films synthesized at 100 °C, 150 °C, and 190 °C over different reaction times.

Section 5. Determination of Optimal Reaction Window from PL Analysis

(a)

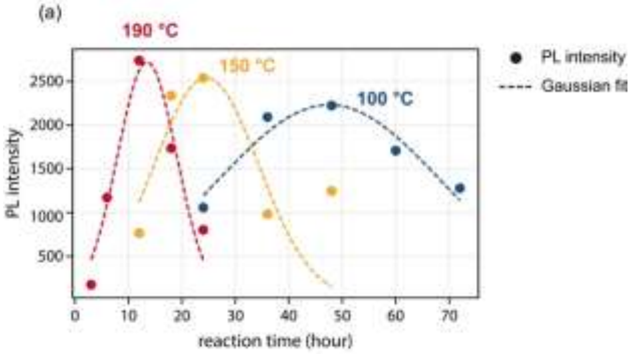

(b)

Temperature	Average (hour)	FWHM (hour)
100 °C	47.6 ± 1.9	49.5 ± 6.2
150 °C	24.6 ± 3.6	23.0 ± 9.0
190 °C	13.5 ± 0.8	13.0 ± 1.8

(c)

Reaction window	100 °C	150 °C	190 °C
**under reaction**	24 hours	12 hours	3-6 hours
**optimal reaction**	36-48 hours	18-24 hours	12 hours
**over reaction**	72 hours	36-48 hours	18-24 hours

Figure S4 (a) PL intensities (markers) as a function of reaction time at 100, 150, and 190 °C with Gaussian fits (dashed lines). (b) Summary table of average reaction times (center values from Gaussian fitting) and corresponding FWHM values (± error). (c) Table summarizing the reaction windows at 100, 150, and 190 °C, showing the corresponding time ranges assigned to the under-, optimal-, and over-reacted regimes.

The morphological and colorimetric changes described in Figure 2 indicate that the optimal condition cannot be regarded as a single point but should instead be defined as an optimal reaction window. Specifically, at 100 °C the optimal condition spans 36–48 hours (a 12 hour window), at 150 °C it spans 18–24 hours (a 6 hour window), whereas at 190 °C the window effectively narrows to a single point at 12 hours.

To substantiate this interpretation quantitatively, we analyzed the PL intensity evolution as a function of reaction time (Figure S4a-b). Gaussian fitting yielded mean optimal times of 47.6 ± 1.9 hours at 100 °C, 24.6 ± 3.6 hours at 150 °C, and 13.5 ± 0.8 hours at 190 °C, consistent

with microscopy-based observations. Accordingly, 48 hours at 100 °C, 24 hours at 150 °C, and 12 hours at 190 °C can be taken as representative optimal points. However, at 100 °C and 150 °C, earlier samples (36 and 18 hours, respectively) still retain ~94% and ~92% of the corresponding maximum intensities, and thus also fall within the optimal reaction window. By contrast, at 190 °C the second-highest intensity (18 hours) drops to ~63% of the highest value, indicating that only 12 hours can represent the optimal condition at this temperature. The corresponding reaction windows–including under-, optimally-, and overreacted regimes for each temperature–are summarized in Figure S4c.

Section 6. Morphological, Chemical, and Vibrational Evolution and Complete Degradation of Mithrene Films at 190 °C

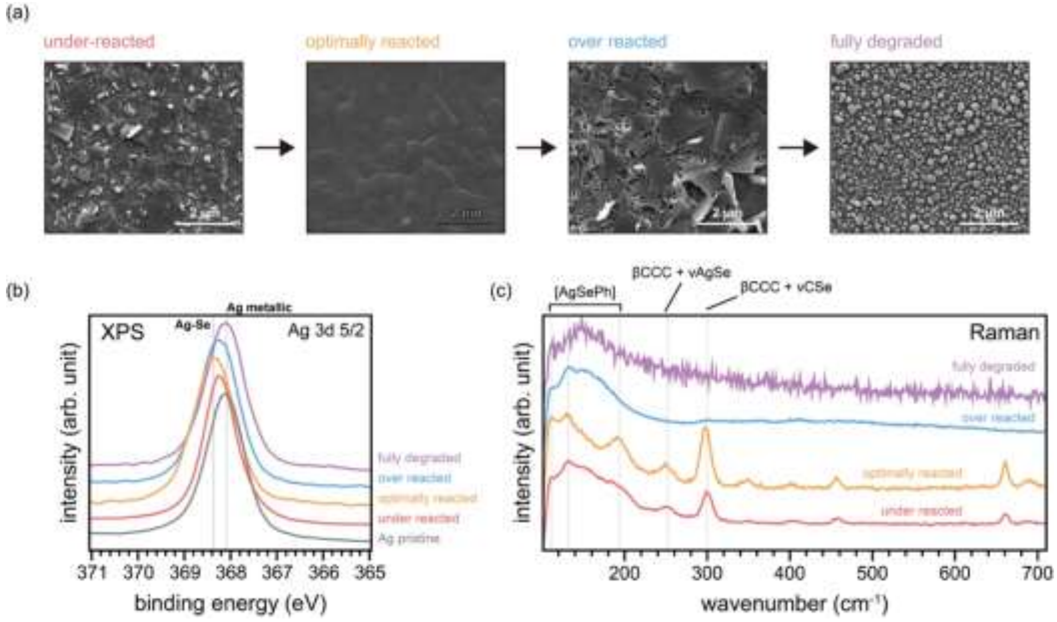

Figure S5 (a) Sequential SEM images showing the morphological evolution of mithrene films synthesized at 190 °C across different stages: under-reacted (3 h), optimally reacted (12 h), over-reacted (24 h), and fully degraded (30 h). (b-c) Overlay of Ag 3d5/2 XPS spectra and Raman spectra for the same stages, respectively.

Figure S5c presents the corresponding Raman spectra, which contains [AgSePh]∞ modes associated with the inorganic planes. In the under-reacted state, these modes appear broad and weak, reflecting incomplete development of the Ag-Se framework. At the optimal condition (12 h), the peaks sharpen and become well resolved, consistent with higher crystallinity and the establishment of an extended [AgSePh]∞ lattice. As the reaction proceeds into the over-reacted state (24 h), the peaks broaden again and lose definition, indicating increasing disorder of the inorganic planes. In the fully degraded state (30 h), all characteristic modes vanish, consistent with the collapse of the [AgSePh]∞ network and the reversion to metallic Ag clusters.

Section 7. Thermal Degradation and Temporal Evolution of Mithrene Films at 200 °C

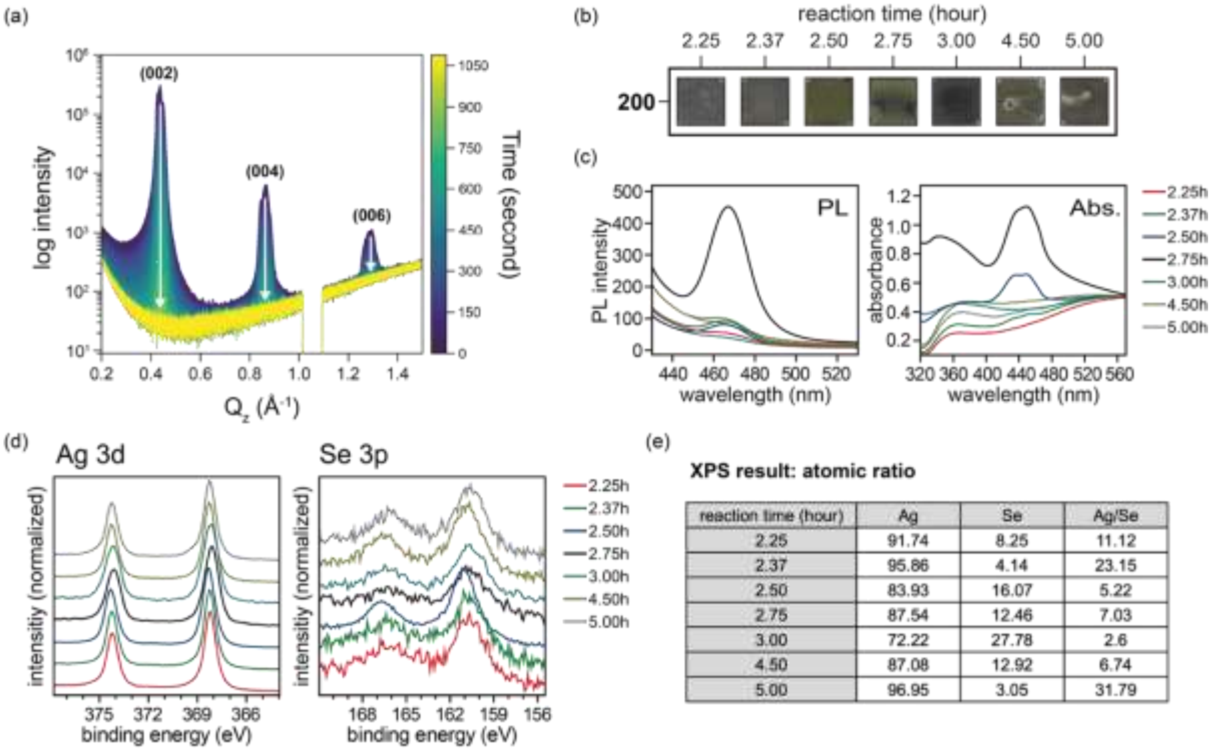

reaction time (hour)	Ag	Se	Ag/Se
2.25	91.74	8.25	11.12
2.37	95.86	4.14	23.15
2.50	83.93	16.07	5.22
2.75	87.54	12.46	7.03
3.00	72.22	27.78	2.6
4.50	87.08	12.92	6.74
5.00	96.95	3.05	31.79

Figure S6 (a) *In situ* GIWAXS result of a mithrene film synthesized at 190 °C for 12 hours (optimal condition), showing the progressive collapse of the layered mithrene structure at 200 °C under high vacuum (~10^{-3} Torr). (b–d) Characterization of mithrene films synthesized directly at 200 °C: (b) optical images at different reaction times, (c) reaction time–dependent PL and UV–vis absorption spectra, and (d) XPS core-level spectra (Ag 3d and Se 3p). (e) Atomic ratios of Ag and Se, and the corresponding Ag/Se ratios, extracted from the XPS analysis in (d).

Section 8. GIWAXS Analysis and Structure Simulation of Mithrene Film

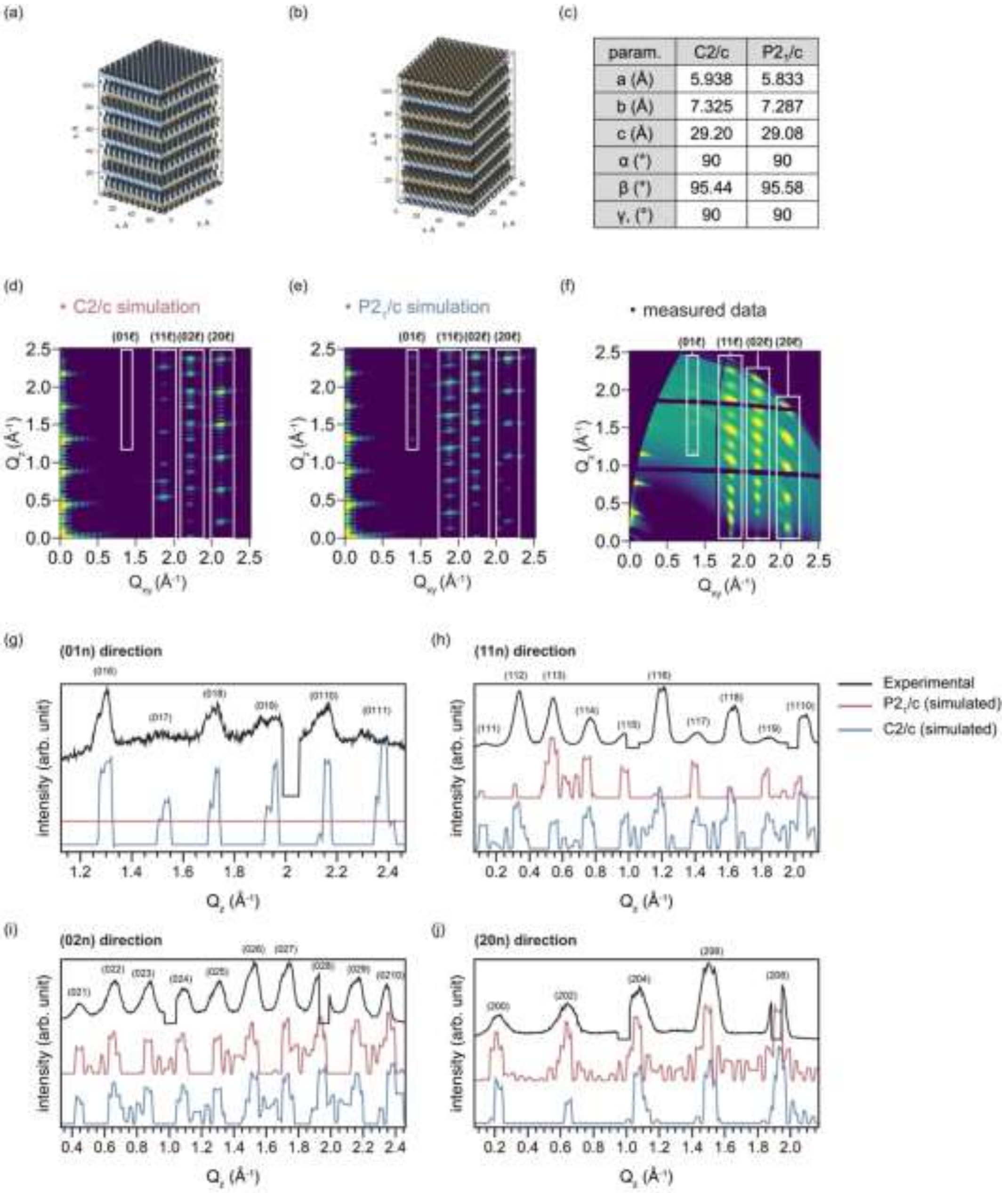

Figure S7 (a-b) Crystal structure models corresponding to the C2/c and P2$_1$/c space groups, respectively. (c) Table summarizing the crystallographic parameters (lattice constants, a, b, c and angles α, β, γ) employed as input for the simulations. (d-e) Simulated GIWAXS patterns based on the C2/c and P2$_1$/c crystal structures, with the preferred orientation set along the (00ℓ) direction; peak broadening due to finite grain size is incorporated in (a–b). (f) The experimentally measured GIWAXS pattern of a mithrene film synthesized at 190 °C for 12 hours, corresponding to the optimal condition at this temperature. In (d-f), the crystallographic

directions (01ℓ), (11ℓ), (02ℓ), and (20ℓ) ($n \in \mathbb{Z}$) are highlighted on the colormaps. (g-j) One-dimensional linecuts along each of these directions in the out-of-plane direction (Q_z) axis, directly compared with the simulated intensities and the experimental data.

A direct comparison between the simulated and experimental GIWAXS linecuts provides clear evidence for the structural assignment of mithrene. Along the (01ℓ) direction, distinct reflections observed experimentally are absent in the C2/c model but are well reproduced by the $P2_1/c$ simulation, including their positions (Figure S7g). Moreover, in the (11ℓ) direction, the C2/c model significantly underestimates reflections, such as the (112) at q ≈ 0.35 $Å^{-1}$, (116) at q ≈ 1.20 $Å^{-1}$, and (118) at q ≈ 1.60 $Å^{-1}$, whereas these features are accurately captured by the $P2_1/c$ simulation. By contrast, the (02ℓ) and (20ℓ) directions (Figures S7i–j) do not show major differences between the two models, and both reproduce the experimental data reasonably well. Consequently, these results clearly demonstrate that mithrene films synthesized via the solvent-free method crystallize in the $P2_1/c$ space group, and the measured data were consistently indexed using the $P2_1/c$ model (Figure S8).

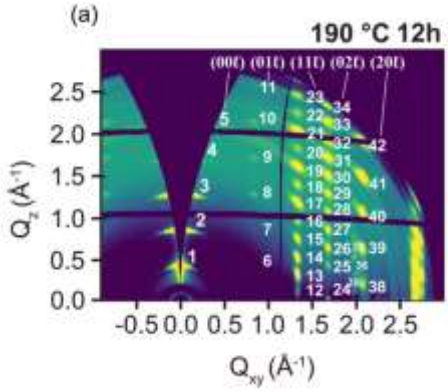

(b)

index number	(hkl)	index number	(hkl)	index number	(hkl)	index number	(hkl)
1	(002)	12	(111)	23	(1112)	34	(0210)
2	(004)	13	(112)	24	(020)	35	(121)
3	(006)	14	(113)	25	(021)	36	(122)
4	(008)	15	(114)	26	(022)	37	(123)
5	(0010)	16	(115)	27	(023)	38	(200)
6	(012)	17	(116)	28	(024)	39	(202)
7	(014)	18	(117)	29	(025)	40	(204)
8	(016)	19	(118)	30	(026)	41	(206)
9	(018)	20	(119)	31	(027)	42	(208)
10	(0110)	21	(1110)	32	(028)		
11	(0112)	22	(1111)	33	(029)		

Figure S8 (a) Index numbers (1–42) are presented on the measured GIWAXS pattern of the mithrene film synthesized at 190 °C for 12 hours. The characteristic crystallographic orientations, including the out-of-plane (00ℓ) and diagonal (01ℓ), (11ℓ), (02ℓ), and (20ℓ) directions, are annotated on the GIWAXS colormap. (b) Table of Miller indices (hkl)

corresponding to each index number, determined by comparison with the simulated pattern based on the $P2_1/c$ structure.

Section 9. Out-of-Plane GIWAXS Profiles and FWHM Analysis

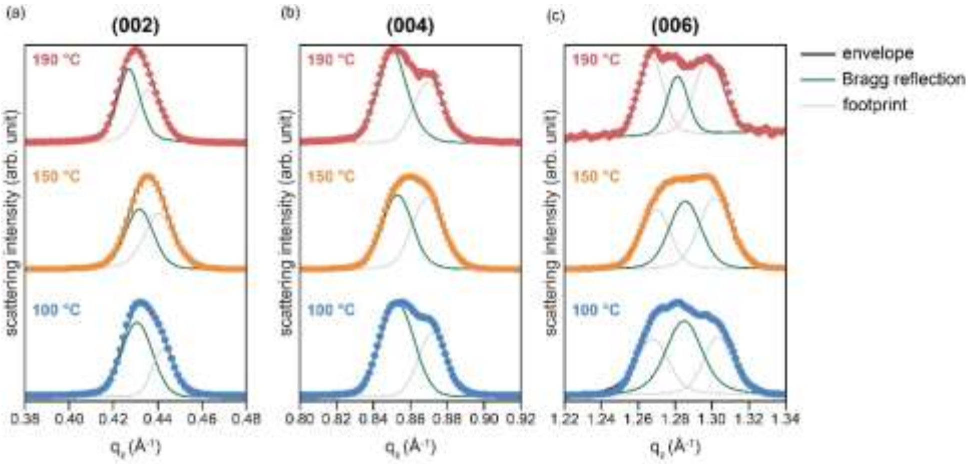

(d)

FWHM ($Å^{-1}$)	100 °C	150 °C	190 °C
(002)	0.0201 ± 0.0006	0.0196 ± 0.0005	0.0178 ± 0.0006
(004)	0.0199 ± 0.0006	0.0191 ± 0.0003	0.0184 ± 0.0006
(006)	0.0275 ± 0.0008	0.0255 ± 0.0007	0.0221 ± 0.0004

Figure S9 (a-c) out-of-plane GIWAXS 1D line profiles of mithrene films synthesized at 100, 150, 190 °C, highlighting the (002), (004), (006) reflections, respectively. (d) Summary table of the extracted full width at half maximum (FWHM) values with fitting errors for each reflection and synthesis temperature.

Section 10. Reaction Temperature-Dependent Orientation Distribution of Solvent-Free Mithrene Films

(a)

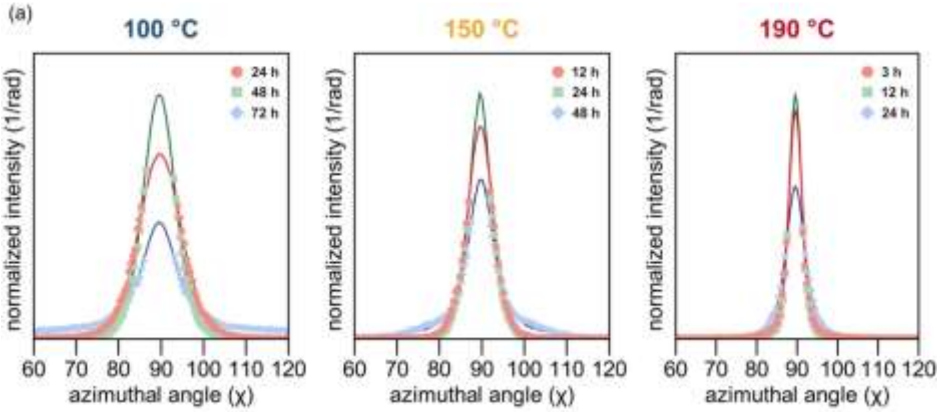

(b)

Reaction time \ Temp.	100 °C	150 °C	190 °C
3h	-	-	6.12 ° ± 0.01 °
12h	-	7.14 ° ± 0.02 °	**3.77 ° ± 0.01 °**
24h	11.94 ° ± 0.03 °	**6.39 ° ± 0.02 °**	6.12 ° ± 0.02 °
48h	**8.99 ° ± 0.02 °**	8.51 ° ± 0.07 °	-
72h	11.81 ° ± 0.11 °	-	-

Figure S10 (a) Orientation distribution functions extracted from the (002) azimuthal intensity profiles of mithrene films at different synthesis temperatures. (b) Full width at half maximum (FWHM) obtained from Voigt-function fitting of the orientation distributions in (a), with fitting uncertainties (± error) included. The optimal condition is highlighted in bold, with 100, 150, and 190 °C data shown in blue, yellow, and red, respectively.

Section 11. Absolute Atomic Percentages from XPS Quantification

(a) 100 °C

reaction time (h)	Ag	Se	$C_{C\text{-}Se}$	$C_{C\text{-}C}$
24	33.89 ± 5.03	9.17 ± 2.50	9.56 ± 1.87	47.38 ± 1.87
36	12.97 ± 1.67	12.89 ± 1.63	12.36 ± 2.49	61.78 ± 2.49
48	12.59 ± 1.76	12.45 ± 1.29	12.49 ± 2.14	62.47 ± 2.14
60	14.13 ± 1.38	12.35 ± 1.62	12.4 ± 1.60	61.12 ± 1.60
72	14.4 ± 1.69	12.24 ± 2.86	12.17 ± 2.35	61.19 ± 2.35

(b) 150 °C

reaction time (h)	Ag	Se	$C_{C\text{-}Se}$	$C_{C\text{-}C}$
12	14.78 ± 5.71	12.12 ± 2.36	12.51 ± 3.84	60.59 ± 3.84
18	12.49 ± 1.73	12.85 ± 1.41	12.44 ± 1.88	62.22 ± 1.88
24	12.65 ± 1.25	12.57 ± 0.90	12.46 ± 0.96	62.32 ± 0.96
36	13.72 ± 1.92	12.38 ± 1.44	12.00 ± 2.19	61.9 ± 2.19
48	15.38 ± 2.68	12.34 ± 1.86	12.06 ± 1.71	60.22 ± 1.71

(c) 190 °C

reaction time (h)	Ag	Se	$C_{C\text{-}Se}$	$C_{C\text{-}C}$
3	31.63 ± 9.16	9.48 ± 2.98	9.85 ± 3.92	49.04 ± 3.92
6	13.81 ± 2.62	11.92 ± 2.34	12.33 ± 2.78	61.94 ± 2.78
12	12.49 ± 1.46	12.46 ± 1.25	12.74 ± 1.51	62.31 ± 1.51
18	13.57 ± 1.72	12.38 ± 2.92	12.18 ± 1.79	61.87 ± 1.79
24	17.66 ± 2.72	12.06 ± 2.39	11.71 ± 1.82	58.57 ± 1.82

Table S2 Absolute atomic percentages of Ag, Se, $C_{C\text{-}Se}$, $C_{C\text{-}C}$ obtained from XPS analysis at (a) 100 °C, (b) 150 °C, and (c) 190 °C as a function of reaction time. For $C_{C\text{–}Se}$ and $C_{C\text{–}C}$, the values include the fitting uncertainties arising from the deconvolution procedure. Sensitivity factors applied for quantification are Ag = 6.277, Se = 0.996, and C = 0.314.

Section 12. XPS Depth Profiling of Optimally Reacted Mithrene Film Synthesized at 190 °C

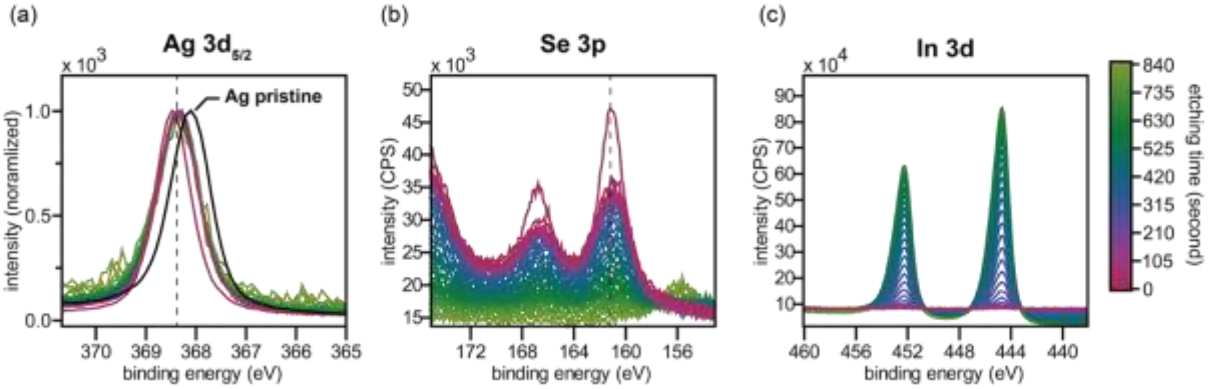

Figure S11 (a-c) X-ray photoelectron spectroscopy (XPS) depth profiling of mithrene film synthesized at 190 °C under the optimal condition: (a) Ag $3d_{5/2}$ region (b) Se 3p region, and (c) In 3d region from the underlying ITO substrate.

Section 13. Second-derivative Analysis of UV-vis Absorption Spectra

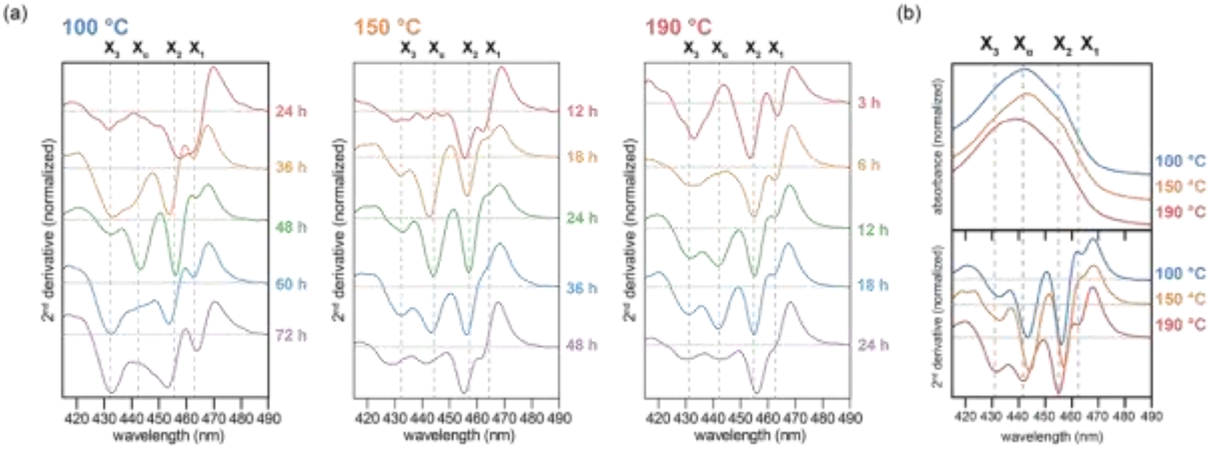

Figure S12 (a) Second-derivative analysis of UV-vis absorption spectra for mithrene films synthesized at 100, 150, 190 °C, highlighting excitonic features (X_1, X_2, X_3, and X_α). (b) Comparison between the original UV-vis absorption spectra and their corresponding second-derivative curves for optimally reacted samples at each temperature.

The horizontal gray line indicates the zero baseline of the second derivative at each reaction time. Owing to spectra overlap from multiple excitonic transitions, the X_1 excitonic feature is not clearly resolved at room temperature.

Section 14. Temporal Evolution of Absorbance Trends at ~464 nm (X_1), ~446 nm (X_α)

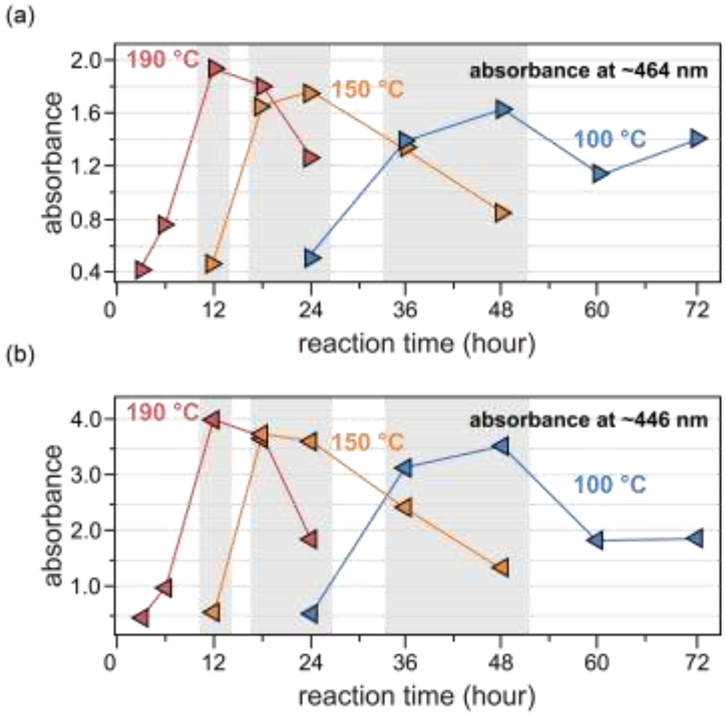

Figure S13 Reaction time-dependent absorbance trends of mithrene films synthesized at 100 °C, 150 °C, 190 °C, measured at excitonic position determined by second-derivative analysis in Figure S8a. (a) Absorbance evolution at ~464 nm, corresponding to the X_1 exciton. (b) Absorbance evolution at ~446 nm, tentatively assigned as X_α.

In both cases (Figure S9a-b), the 190 °C samples exhibit the highest absorbance at the optimal reaction time, followed by 150 °C and 100 °C. However, the reaction window narrows with increasing temperature.

Section 15. Temperature and pressure measurements in conventional glass vial setups under air (solvent-free) and air + solvent conditions at 100 °C

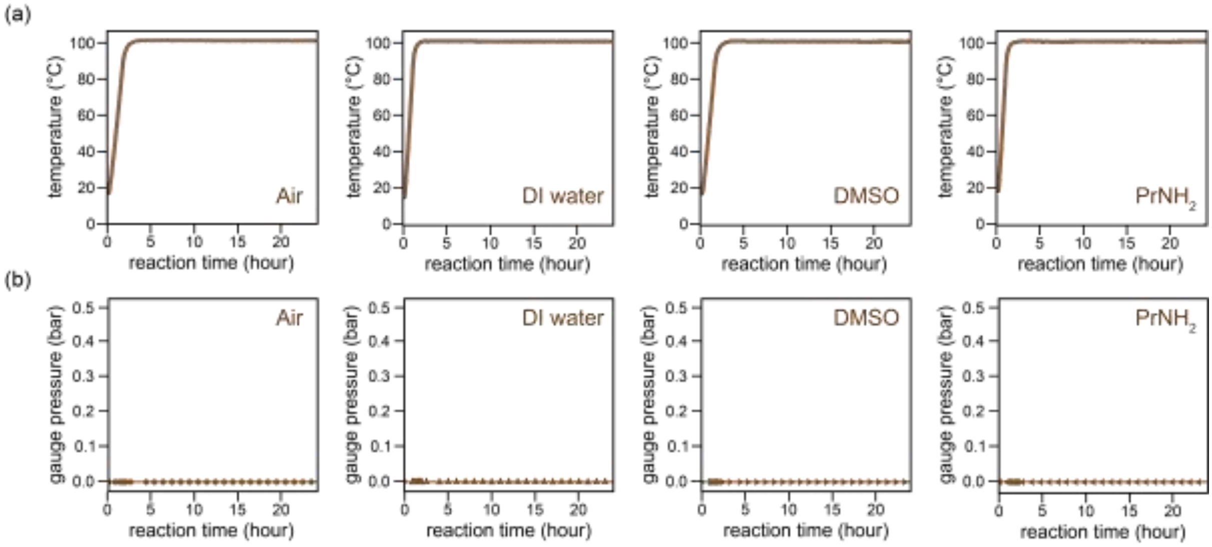

Figure S14 (a) Internal temperature profiles measured during the reaction in conventional glass vial setups containing air without solvent, air + DI water, air + DMSO, and air + $PrNH_2$, all reaching 100 °C. (b) Corresponding internal gauge pressure measurements, showing 0 bar in all cases despite reaching target temperatures.

Section 16. Thermal and Pressure Profiles in the Reaction Chamber under Air and Air + Solvent Conditions at 100 °C

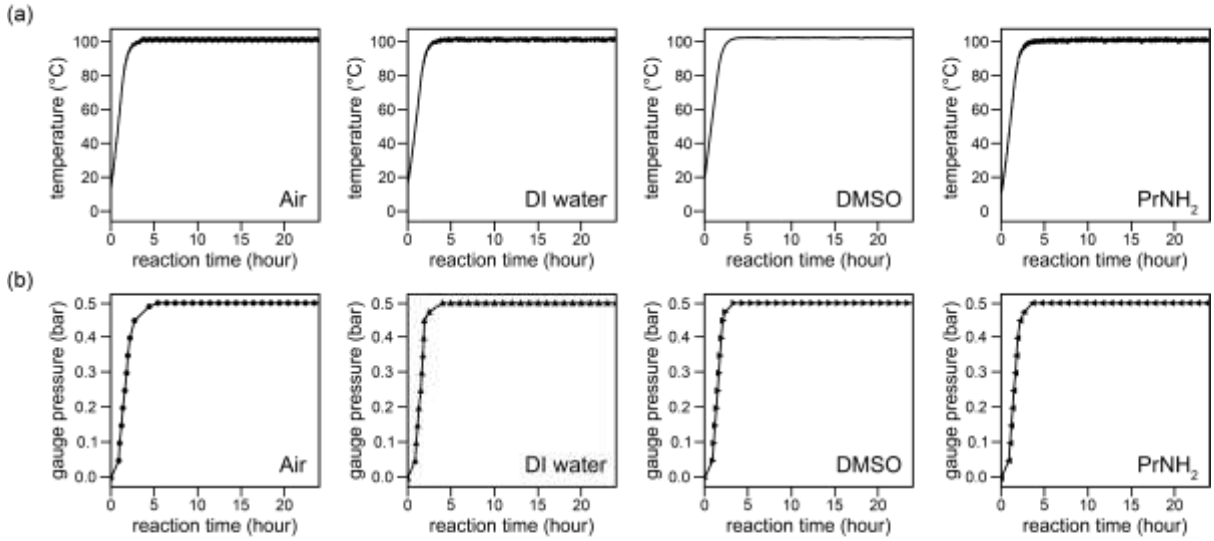

Figure S15 (a) Internal temperature profiles measured during the reaction in the reaction chamber setups containing air without solvent, air + DI water, air + DMSO, and air + $PrNH_2$, all reaching 100 °C. (b) Corresponding internal gauge pressure measurements, showing 0.5 bar, confirming proper sealing and pressure retention in the chamber.

Section 17. XPS Analysis of Se 3p and Ag 3d Core Levels of Mithrene Films Synthesized under Different Atmosphere

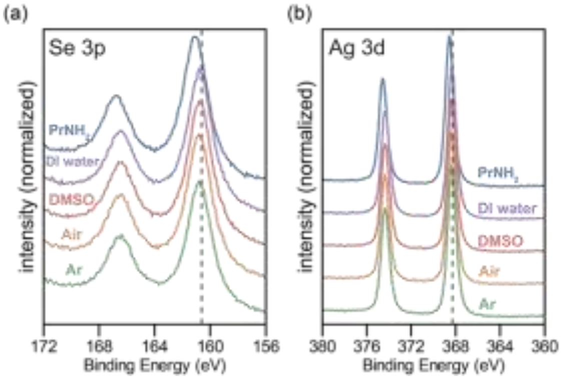

(c)

Atomic ratio	Ag 3d %	Se 3p %
Ar without solvent	50.0	50.0
Air without solvent	49.1	50.9
Air with DMSO	48.8	51.2
Air with DI water	48.9	51.1
Air with $PrNH_2$	48.8	51.2

Figure S16 (a) Se 3p and (b) Ag 3d core-level XPS spectra of mithrene films synthesized under different atmospheric conditions: Ar without solvent, air without solvent, and air with DMSO, DI water, and $PrNH_2$. (c) Atomic ratios of Ag and Se extracted from XPS analysis.

Films synthesized under Ar without solvent exhibited an ideal stoichiometry (50.0:50.0), while films prepared under air-based conditions showed increasing deviations from the ideal ratio (air without solvent: 49.1:50.9, air with DMSO: 48.8:51.2; air with DI water: 48.9: 51.1, air with $PrNH_2$: 48.8:51.2), indicating the detrimental effect of atmospheric exposure and solvent use on stoichiometric control

Section 18. Second-derivative Spectra Analysis and Absorbance Trends of Solvent-Free and Solvent-Assisted Samples

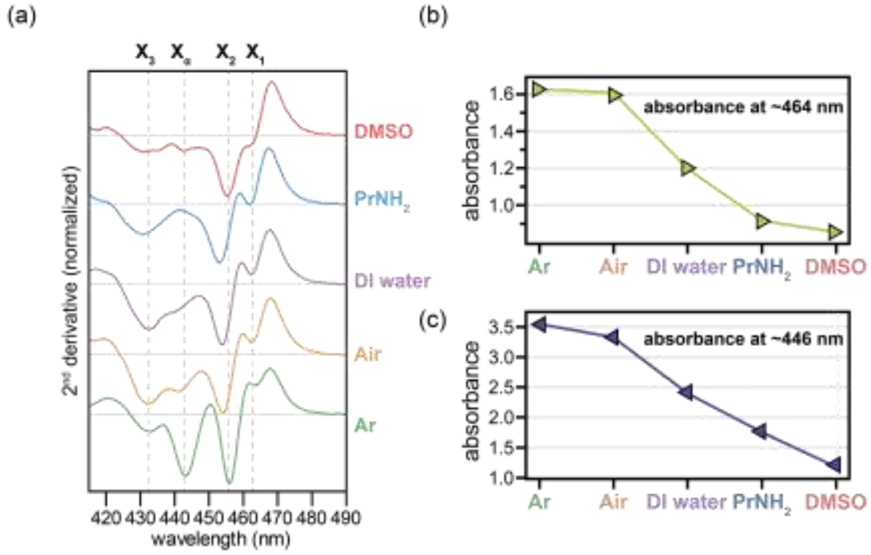

Figure S17 (a) Second-derivative analysis of UV-vis absorption spectra for mithrene films synthesized under Ar without solvent, air without solvent, air + DI water, air + DMSO, and air + $PrNH_2$. (b) Absorbance evolution at ~464 nm, corresponding to the X_1 exciton. (c) Absorbance evolution at ~446 nm, tentatively assigned as X_α.

The analysis resolves distinct excitonic features including X_1, and X_α positions for each sample, showing that Ar without solvent exhibits the strongest absorption, consistent with its superior structural quality

Section 19. Characterization of Mithrene Films Synthesized under N_2 Atmosphere

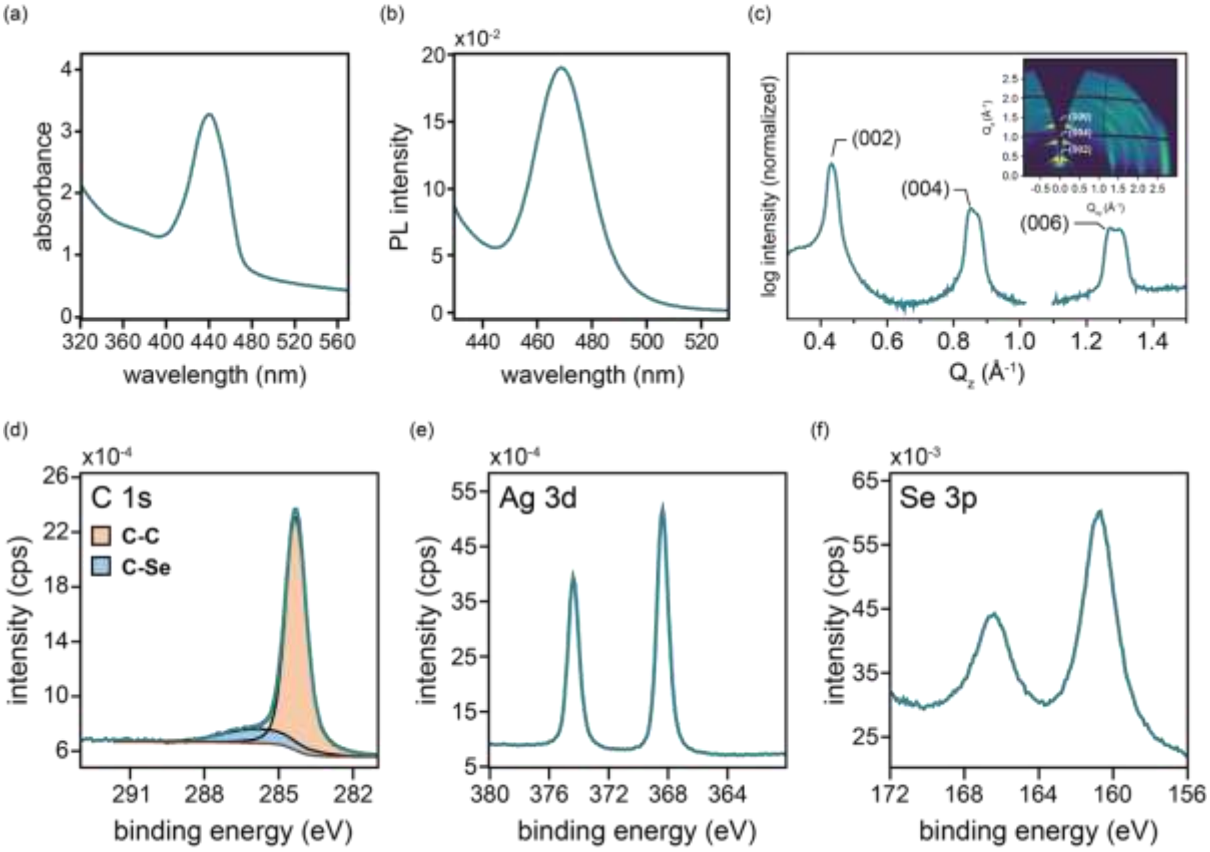

Figure S18 (a-b) UV-vis absorption, photoluminescence spectra (c) GIWAXS out-of-plane 1d profile, with corresponding 2D GIWAXS map inset. (d) XPS C 1s core-level spectrum showing deconvoluted peaks for $C_{C\text{-}C}$ (~284.6 eV) and $C_{C\text{-}Se}$ (~286.0 eV). (e,f) XPS spectra of Ag 3d and Se 3p regions, respectively.

The reaction chamber was fully assembled inside a nitrogen-filled glove box (moisture, and oxygen levels < 0.01 ppm), where the Ag-coated substrate and 35 mg of DPSe powder were loaded under inert conditions. After final sealing to ensure complete atmospheric isolation, the chamber was transferred to a laboratory oven and heated at 100 °C for 48 hours to carry out the reaction.

To evaluate the broader applicability of our solvent-free approach, we further tested mithrene synthesis under a nitrogen (N_2) atmosphere using the same stainless-steel chamber. As shown

in Figure S18, the resulting films exhibit comparable optical and structural characteristics to those synthesized under argon. The UV-vis absorption and PL spectra revealed well-defined excitonic features, while GIWAXS confirms that the absence of misaligned domains in the layered mithrene structure. XPS analysis showed a relative atomic ratio is equal to 1.00:0.99:1.02, verifying the formation of stoichiometric AgSePh. These results demonstrate that the optimized reaction kinetics achieved in an inert atmosphere are not limited to Ar, but are also compatible with other inert gas, highlighting its versatility and robustness of this method for solvent-free MOC film fabrication.

Section 20. Comparative Structural, Chemical, and Optical Properties under Different Reaction Conditions

Condition	(002) Azimuthal FWHM (°)	Oxygen atomic ratio (%)	PL Intensity	Abs. X_2	Abs. X_α	Abs. X_3
Ar	8.54	0.0	1784.5	2.49	3.55	3.42
Air	9.55	1.66	1238.3	2.42	3.33	3.28
Air + DI water	11.49	1.22	1356.4	1.81	2.4	2.43
Air + $PrNH_2$	10.61	2.40	866.2	1.36	1.75	1.8
Air + DMSO	12.37	0.0	1168.1	1.11	1.18	1.09

Table S3 Summary of quantitative parameters for mithrene films synthesized under different environments: (002) azimuthal FWHM, XPS O 1s oxide fraction, PL intensity, and absorbance values at X_2, X_α, and X_3.

Table S3 provides a consolidated comparison of structural, chemical, and optical properties of mithrene films prepared in different environments. From the GIWAXS analysis, the solvent-free Ar condition yields the narrowest (002) azimuthal FWHM. In the XPS O 1s spectra (Figure 6e), oxide-related components are absent in samples synthesized under Ar, confirming their chemical integrity. Optical measurements further confirm the advantages of the Ar condition, as this sample exhibits the highest PL intensity as well as strongest absorbance features at X_2, X_α, X_3.

Reference

(1) Lee, W. S.; Cho, Y.; Powers, E. R.; Paritmongkol, W.; Sakurada, T.; Kulik, H. J.; Tisdale, W. A. Light Emission in 2D Silver Phenylchalcogenolates. *ACS Nano* **2022**, *16* (12), 20318–20328. DOI: 10.1021/acsnano.2c06204.

(2) Hohman, J. N. Tarnishing Silver Metal into Mithrene. *JACS* **2018.** DOI: 10.1021/jacs.8b08878

(3) Maserati, L.; Pecorario, S.; Prato, M.; Caironi, M. Understanding the Synthetic Pathway to Large-Area, High-Quality $[AgSePh]_\infty$ Nanocrystal Films. *The Journal of Physical Chemistry C* **2020**, *124* (41), 22845–22852. DOI: 10.1021/acs.jpcc.0c07330.

(4) Tisdale, W. A. Morphological Control of 2D Hybrid Organic− Inorganic Semiconductor AgSePh. *ACS Nano* **2022.** DOI: 10.1021/acsnano.1c07498

(5) Yang, G.; Liu, Y.; Wang, T.; Wu, Y.; Wang, J. Area-scalable film preparation and blue excitonic photoluminescence of organic–inorganic hybrid 2D semiconductor AgSePh (Ph = C_6H_5). *Dalton Transactions* **2025**, *54* (34), 12970–12978. DOI: 10.1039/d5dt01290b.

(6) Schriber, E. A.; Popple, D. C.; Yeung, M.; Brady, M. A.; Corlett, S. A.; Hohman, J. N. Mithrene Is a Self-Assembling Robustly Blue Luminescent Metal–Organic Chalcogenolate Assembly for 2D Optoelectronic Applications. *ACS Applied Nano Materials* **2018**, *1* (7), 3498–3508. DOI: 10.1021/acsanm.8b00662.
